# Telomere integrity, epigenetic aging, and genetic burden shape biological aging trajectories in idiopathic pulmonary fibrosis

**DOI:** 10.1038/s41514-026-00434-1

**Published:** 2026-07-03

**Authors:** Manuela Campisi, Luana Cannella, Filippo Liviero, Federico Tamiazzo, Elisabetta Balestro, Paolo Spagnolo, Sofia Pavanello

**Affiliations:** 1 Department of Cardiac, Thoracic and Vascular Science and Public Health- DSCTV, University of Padua, Padua, Italy; 2 University—Hospital of Padova, Padova, Italy; 3 BMR Genomics, via Redipuglia 21/A—35131, Padova, Italy; 4 Centre of Studies and Activities for Space CISAS “G. Colombo” of University of Padua, Padua, Italy

**Keywords:** Biomarkers, Diseases, Genetics, Medical research, Molecular biology, Molecular medicine

## Abstract

Idiopathic pulmonary fibrosis (IPF) is a paradigmatic aging-related lung disorder. In this retrospective cohort study, we evaluated 101 treatment-naïve patients at diagnosis (T0) and a subgroup (*n* = 31) after one year of antifibrotic therapy (T1). Analyses included leukocyte telomere length (LTL), DNA methylation age [DNAmAge assessed by Horvath, Levine (PhenoAge), Skin & Blood, Hannum, BLUP, Elastic Net (EN), and a 5-CpG panel], age acceleration (AgeAcc), and genetic susceptibility. At T1, LTL was independently predicted by baseline LTL (*p* = 0.0004) and treatment duration (*p* = 0.0056). Longitudinally, ΔLTL increased in nintedanib- versus pirfenidone-treated patients (*p* = 0.0402) and with treatment duration (*p* = 0.0233). DNAmAge modestly increased with chronological aging across all clocks, while AgeAcc remained stable, decreasing at follow-up (*p* = 0.0435) and higher in males (*p* = 0.0204). Genetic analyses on 17 IPF-associated SNPs confirmed enrichment of established risk variants, including *MUC5B* and *DPP9*, and identified an association between higher genetic burden and lower forced vital capacity (*p* = 0.0136). Extending this approach, genome-wide imputation enabled polygenic risk score (PRS) analysis, revealing significant case–control differences and discrimination (AUC up to 0.79), supporting a measurable polygenic contribution to disease susceptibility. These findings highlight the added value of integrating telomeric, epigenetic, and genome-wide genetic burden—captured through PRS—for improved risk stratification in IPF.

## Introduction

Aging represents a major global health challenge with profound societal and economic implications, driving chronic diseases, performance status decline, and premature mortality^1^. Yet, aging is far from uniform: while some individuals maintain physiological resilience, others undergo accelerated biological aging leading to early dysfunction. This heterogeneity highlights the limitations of chronological age as a predictor of health. Biological aging—the cumulative burden of molecular damage and systemic dysregulation—offers a more precise and clinically relevant measure^2^. Among its central mechanisms are telomere attrition and epigenetic drift^3^.

Within this framework, Idiopathic Pulmonary Fibrosis (IPF) emerges as a paradigmatic model to study biological aging. IPF is a rare, chronic and progressive interstitial lung disease primarily affecting older adults, characterized by fibrotic remodeling, dyspnea, and progressive loss of respiratory function^4,5^, affecting ~3 million people worldwide^5^. It is the most common form among all different interstitial lung diseases, with incidence increasing—partly due to population aging and possibly post-COVID-19 fibrotic sequelae^6^. The prognosis remains poor: median survival is ~3 years, and the disease imposes high healthcare costs^4^ and a substantial psychosocial burden^7^.

Although defined as “idiopathic,” the pathogenesis of IPF reflects the interplay of multiple risk factors. Aging represents a major determinant^8^, but cumulative exposures and environmental insults also play a key role. Robust evidence from systematic reviews and meta-analyses highlights occupational exposures—particularly to metal, wood, silica/stone, and agricultural or organic dusts, including livestock—as strong contributors to disease risk^9–12^. Environmental pollutants, notably air pollution, further exacerbate susceptibility^10–14^. Other exposures—including pesticides, hairdressing-related chemicals, bird breeding, molds, textile dust, and fire smoke—have also been reported, although their associations with IPF are less consistent across studies^10,11^. Lifestyle determinants such as cigarette smoking and dietary habits, together with genetic predisposition, add to this complex risk profile^12^.

IPF is also the most frequent clinical manifestation of telomere-mediated disease, directly linking it to mitotic aging mechanisms^15–17^. Telomeres—repetitive DNA sequences protecting chromosomal integrity—shorten with each cell division. Telomere attrition, measurable through leukocyte telomere length (LTL), represents a recognized biomarker of biological age and systemic stress^18,19^. Genetic susceptibility further shapes IPF risk. Genome-wide association studies (GWAS) have identified variants in genes involved in telomere maintenance *(TERT, TERC, OBFC1*^20,21^*)*, epithelial integrity *(MUC5B, DSP)*, immune regulation *(DPP9)*, and fibrotic remodeling *(FAM13A, ATP11A)*. Among these, the MUC5B rs35705950 promoter polymorphism confers a four-fold increased risk of IPF, particularly in Europeans, while shorter LTL predicts worse clinical outcomes^15,22,23^. Strikingly, epigenetic inheritance of shortened telomeres has been observed even without causal mutations, suggesting non-Mendelian transmission mechanisms in IPF^24^.

Alongside telomere biology, epigenetic mechanisms—especially DNA methylation age (DNAmAge)—capture non-mitotic aspects of biological aging. DNAmAge, derived from methylation patterns at selected CpGs, correlates strongly with chronological age^25,26^. Accelerated epigenetic aging (AgeAcc)—when DNAmAge exceeds chronological age—has been linked to frailty, multimorbidity, and mortality^27–32^. In IPF, evidence on epigenetic aging remains limited. A pioneering study by Kurbanov et al.^33^ reported tissue-specific epigenetic age acceleration in lung samples from a small IPF cohort. However, DNAmAge has not yet been systematically investigated in peripheral blood, nor integrated with LTL, genetic susceptibility, or longitudinal treatment effects, leaving the systemic aging profile of IPF largely unexplored.

Therapeutically, nintedanib and pirfenidone remain the only approved antifibrotic agents. They slow functional decline but are not curative^34,35^. Nintedanib inhibits receptor tyrosine kinases that drive fibroblast activation^36^, whereas pirfenidone modulates TGF-β signaling and attenuates oxidative stress^37^, potentially limiting telomere shortening and delaying senescence. By intersecting with aging-related pathways, these agents reinforce the need to integrate biological aging biomarkers into IPF clinical monitoring and therapeutic development.

The hallmark-based decomposition analyses—linking diseases to the 12 hallmarks of aging—place IPF among the most aging-aligned conditions^38^. IPF disrupts multiple hallmarks early, including telomere attrition, senescence, deregulated nutrient sensing, genomic instability, and mitochondrial dysfunction^39,40^. This makes IPF a unique model for investigating biological aging mechanisms and testing geroprotective strategies.

To better understand the contribution of biological aging to IPF pathogenesis and prognosis, we adopted a multimodal approach integrating clinical, molecular, and genetic data. LTL was as assessed at diagnosis (T0) in a well-characterized cohort of treatment-naïve IPF patients with clinical outcomes, pulmonary function, hematochemical parameters and occupational exposures. Seven DNA methylation-based epigenetic clocks (Horvath, Levine (PhenoAge), Horvath Skin & Blood, Hannum, Best Linear Unbiased Prediction (BLUP), Elastic Net (EN) and a targeted 5-CpG panel) were applied to evaluate methodological consistency and feasibility, with the aim of identifying a minimal, robust CpG panel for future NGS-based clinical applications. Biological aging biomarkers—LTL, DNAmAge, and AgeAcc—were further examined longitudinally after one year of treatment (T1). In parallel, selected IPF patients were genotyped for known susceptibility variants to support genetic stratification and refine disease risk assessment.

Through this integrated framework, we sought to elucidate the role of biomarkers of biological aging in IPF pathogenesis and progression, and to explore their potential as predictive tools to inform precision medicine strategies.

## Results

### Characteristics of IPF patients

Table 1 summarizes characteristics of IPF patients at enrollment (T0). The majority were male (83.2%) with a mean age of 69 ± 9 years and LTL at T0 averaged 1.27 ± 0.34 (T/S). Nonparametric linear regression analysis revealed no significant correlation between LTL and patient age at T0 (*p* = 0.780). Multiple linear regression analysis did not identify any significant correlations between LTL and various demographic and clinical variables (Supplementary Table 1).

**Table 1:** Characteristics of IPF patients (*n* = 101) at diagnosis (T0)

Variables	Mean ± SD	Number (%)
Gender (male %)		84 (83)
Age (years)	69.0 ± 9.00	
BMI (kg/m^2^)	26.0 ± 4.00	
Pack years ((cigarettes/20)×years)	21.0 ± 23.0	
Leukocytes (10^3^/ml)	7.94 ± 3.55	
Neutrophils (10^3^/ml)	5.00 ± 2.00	
% Neutrophils	58.0 ± 8.00	
Lymphocytes (10^3^/ml)	2.30 ± 0.75	
% Lymphocytes	30.0 ± 8.00	
Monocytes (10^3^/ml)	0.67 ± 0.20	
% Monocytes	8.52 ± 2.21	
FVC (L)	2.61 ± 0.73	
FVC (% pred)	76.0 ± 20.0	
Therapy		
No therapy		4 (4)
Nintedanib		39 (39)
Pirfenidone		58 (57)
LTL	1.27 ± 0.34	

*BMI* body mass index, *FVC* forced vital capacity, *FVC (%pred)* forced vital capacity (percentage of the predicted normal value).

### Characteristic and biological aging markers of follow-up

As reported in Table 2, a total of 31 IPF patients underwent the follow-up assessment after a median time of 310 days of antifibrotics treatment (T1). The majority were male (77.4%), with a mean chronological age of 70.9 ± 7.10 years. At follow-up (T1), a slight, non-significant increase in LTL was observed compared with baseline (T0) (p = 0.458, Table 2).

**Table 2:** Main characteristics of *n* = 31 IPF patients at follow-up (T1)

Variables	Mean ± SD	Number (%)
Gender (Males %)		24 (77.4)
Age (years)	70.9 ± 7.10	
BMI (Kg/m^2^)	26.9 ± 3.65	
Pack-years ((cigarettes/20)×years)	22.3 ± 21.8	
Leukocytes (10^3^/ml)	11.6 ± 14.3	
Neutrophils (10^3^/ml)	4.92 ± 1.17	
% Neutrophils	58.7 ± 7.08	
Lymphocytes (10^3^/ml)	2.17 ± 0.65	
% Lymphocytes	29.2 ± 6.47	
Monocytes (10^3^/ml)	0.63 ± 0.18	
% Monocytes	8.23 ± 1.90	
Occupational risk factor		6 (19.35)
Months from diagnosis to therapy	3.42 ± 7.09	
Follow-up (days of treatment)	310.6 ± 110.4	
Decline (FVC %pred T1–T0)	–3.02 ± 7.35	
LTL at beginning of follow-up (T0)	1.19 ± 0.26	
LTL at end of follow-up (T1)	1.23 ± 0.27	
5-CpG panel DNAmAge at beginning of follow-up (T0)	60.42 ± 6.94	
5-CpG panel DNAmAge at end of follow-up (T1)	60.42 ± 6.44	
5-CpG panel AgeAcc at beginning of follow-up (T0)	–9.55 ± 3.91	
5-CpG panel AgeAcc at end of follow-up (T1)	–10.45 ± 4.22	

*BMI* body mass index, *FVC (%pred)* forced vital capacity (percentage of the predicted normal value), *LTL* leukocyte telomere length, *DNAmAge* DNA methylation age, *AgeAcc* age acceleration.

Multiple linear regression analysis (Supplementary Table 2), including BMI, gender, pack-years, decline in lung function (FVC%pred. T1–T0), age at baseline (T0), baseline values of LTL, treatment duration, and occupational risk factors, indicated that LTL at T1 was significantly associated with both treatment duration (*p* = 0.0056) and baseline LTL (*p* = 0.0004), as highlighted in Table 3.

**Table 3:** Significant results of multiple regression analyses evaluating the influence of BMI (Kg/m^2^), gender (M = 1; F = 0), pack-years ((cigarettes/20) x years), lung function decline (FVC%pred T1-T0), age at beginning of follow-up T0 (years), LTL / 5-CpG panel DNAmAge/ AgeAcc at (T0), duration of treatment (days), and occupational risk factor on LTL, 5-CpG panel DNAmAge and AgeAcc at follow up (T1)

	Variables	b	r	t	*P* value
***T1 LTL***	LTL T0	b6 = 0.642611	r = 0.66351	t = 4.159686	*P* = 0.0004
	Duration of treatment (days)	b7 = 0.001157	r = 0.547459	t = 3.068487	*P* = 0.0056
*T1 5-CpG panel DNAmAge*	Gender (M = 1)	b2 = 2.083691	r = 0.401535	t = 2.056428	*P* = 0.0518
	5-CpG panel DNAmAgeT0	b6 = 0.791651	r = 0.860308	t = 7.915657	*P* < 0.0001
T1 5-CpG panel AgeAcc^*^	Gender (M = 1)	b2 = 2.826082	r = 0.493077	t = 2.718107	*P* = 0.0123
	5-CpG panel AgeAcc T0	b5 = 0.861377	r = 0.871537	t = 8.524344	*P* < 0.0001

^*^The variable Age is not considered for AgeAcc because of its own definition.

*LTL* leukocyte telomere length, *DNAmAge* DNA methylation age, *AgeAcc* age acceleration.

Further regression analysis on longitudinal changes (Supplementary Table 3) including BMI, gender, antifibrotic therapy (0 = pirfenidone; 1 = nintedanib), pack-years, age at baseline, lung function decline (FVC%pred T1-T0), and duration of treatment (days) on **Δ**LTL (LTL T1-T0) revealed that the increase in ΔLTL was significantly greater in patients treated with nintedanib compared with those receiving pirfenidone (*p* = 0.0402) and showed a positive association with treatment duration (*p* = 0.0233), as highlighted in Table 4.

**Table 4:** Significant results of multiple regression analyses evaluating the influence of BMI (Kg/m^2^), gender (M = 1; F = 0), antifibrotic therapy (0 = pirfenidone; 1 = nintedanib), pack-years ((cigarettes/20)×years), age at T0 (years), lung function decline (FVC%pred T1-T0), and duration of treatment (days) on *Δ*LTL (LTL T1-T0) and Δ5-CpG panel AgeAcc (5-CpG panel AgeAcc T1-T0)

	Variables	b	r	t	*P* value
***ΔLTL***	Therapy (*P* = 0; N = 1)	b3 = 0.197156	r = 0.413044	t = 2.175101	*P* = 0.0402
	Duration of treatment (days)	b7 = 0.00093	r = 0.451936	t = 2.429691	*P* = 0.0233
Δ5-CpG panel AgeAcc*	Gender (M = 1)	b2 = 2.621248	r = 0.45209	t = 2.483017	*P* = 0.0204

^*^The variable Age is not considered for AgeAcc because of its own definition.

*LTL* leukocyte telomere lenght.

Epigenetic age estimation and longitudinal changes: DNAmAge levels estimated using the 5-CpG panel and multiple Illumina MethylationEPIC array–based clocks (Horvath, Levine (PhenoAge), Skin & Blood, Hannum, BLUP, and EN) were compared between baseline (T0) and follow-up (T1) after antifibrotic treatment in a subgroup of 27 IPF patients.

**Fig. 1:**
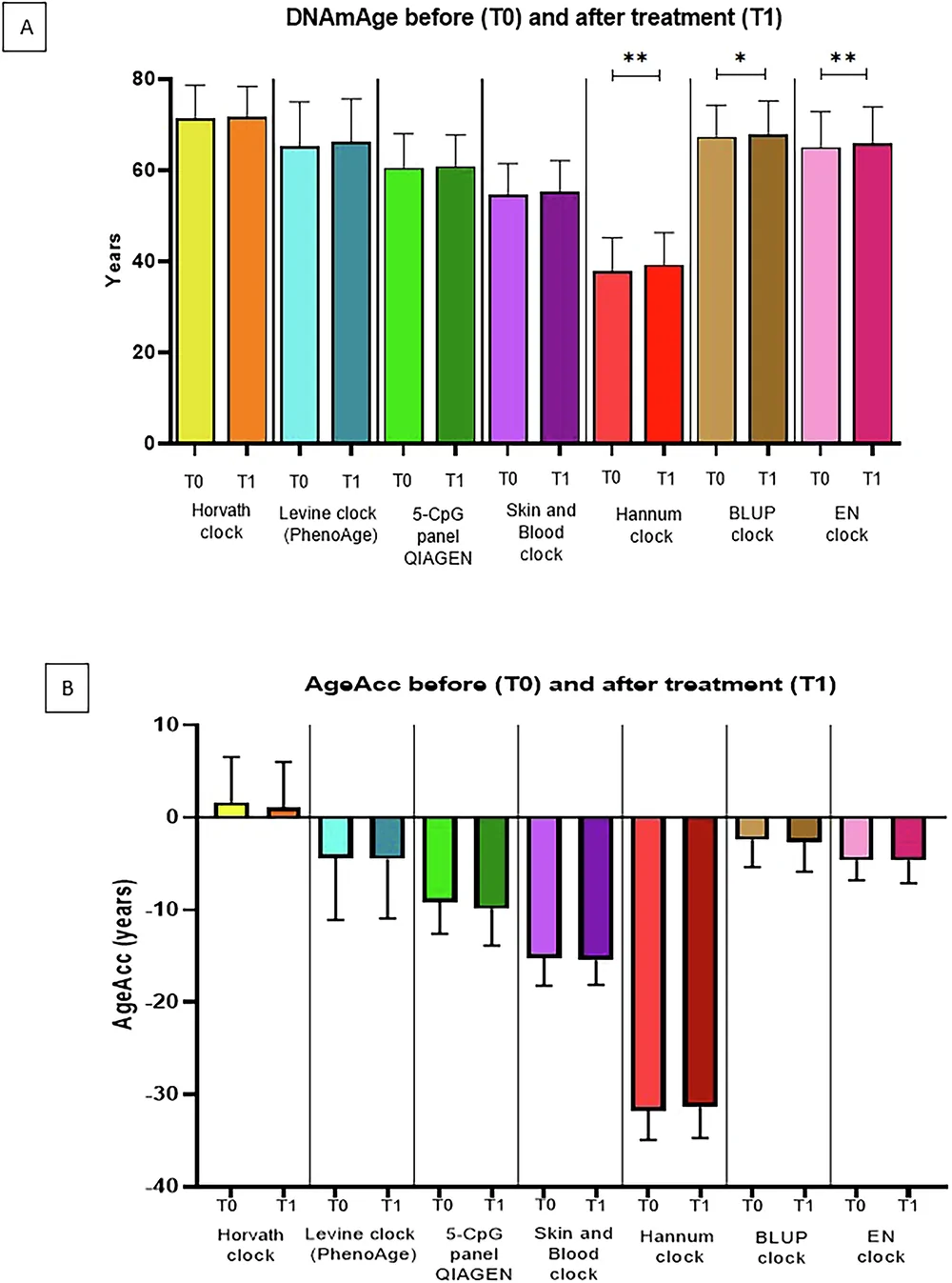
DNAmAge in IPF patients before (T0) and after (T1) antifibrotic treatment, estimated using a 5-CpG panel Qiagen system and Illumina MethylationEPIC array (Horvath, Levine, Horvath Skin & Blood, Hannum, BLUP and EN clocks), and AgeAcc estimated as difference between DNAmAge and chronological age. **A** Bar plots illustrate DNAmAge estimates obtained using seven distinct methods: the Horvath (yellow), Levine (blue), Horvath Skin & Blood (purple), Hannum (red), BLUP (brown), and EN (pink) clocks applied to Illumina methylation array, and the 5-CpG custom methylation panel (green). For each method, DNAmAge at baseline (T0) and after antifibrotic treatment (T1) is shown. No significant differences in DNAmAge were observed between T0 and T1 for the Horvath, Levine, 5-CpG panel Qiagen, and Horvath Skin & Blood clocks (*p* > 0.05). Significant differences between T0 and T1 were observed for the Hannum (*p* = 0.0048), BLUP (0.0148), and EN (0.0030) clocks. **B** Bar plots illustrate AgeAcc estimates as difference between DNAmAge and chronological age. DNAmAge was obtained using seven distinct methods: the Horvath (yellow), Levine (blue), Horvath Skin & Blood (purple), Hannum (red), BLUP (brown), and EN (pink) clocks applied to Illumina methylation array, and the 5-CpG custom methylation panel (green). For each method, AgeAcc at baseline (T0) and after antifibrotic treatment (T1) is shown. No significant differences in AgeAcc were observed between T0 and T1 (*p* > 0.05).

As shown in Fig. 1A, no statistically significant changes in DNAmAge were observed between T0 and T1 for the Horvath, Levine, 5-CpG panel, and Skin & Blood clocks (all *p* > 0.05). In contrast, modest but statistically significant increases in DNAmAge were detected for the Hannum, BLUP, and EN clocks (*p* = 0.0048, *p* = 0.0148, and *p* = 0.0030, respectively), likely reflecting methodological differences in CpGs selection and sensitivity to chronological aging rather than treatment-related effects. Consistently, no significant differences were observed in age acceleration (AgeAcc) between T0 and T1 across all clocks (Fig. 1B), supporting the absence of a measurable treatment effect on epigenetic aging trajectories.

Detailed DNAmAge and AgeAcc values, including mean ± SD and paired comparisons, are reported in Supplementary Table 4 (DNAmAge) and Supplementary Table 5 (AgeAcc).

Correlation between chronological age and DNAmAge: The relationship between chronological age and DNAmAge was evaluated across all clocks in both controls and IPF patients.

**Fig. 2:**
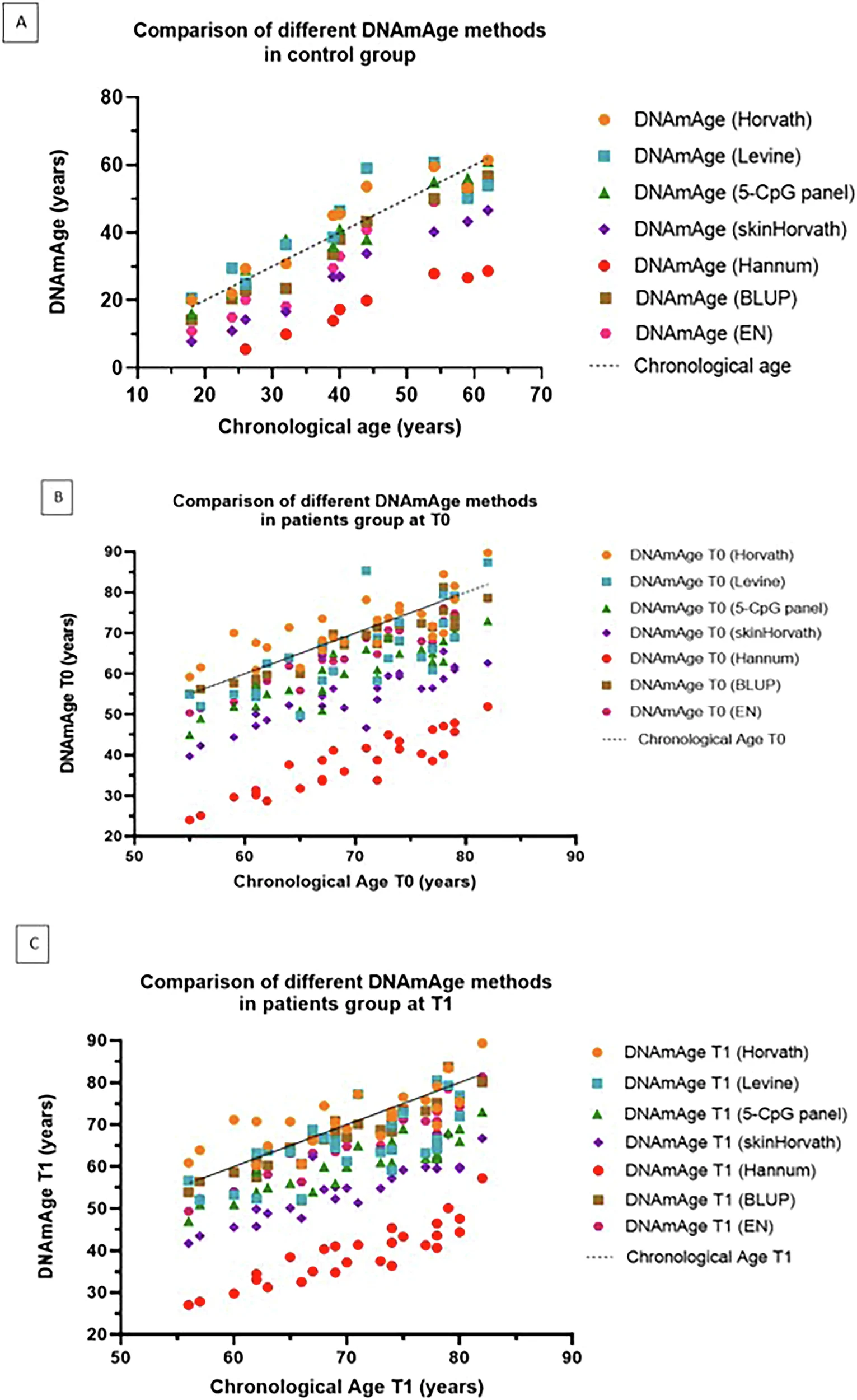
Correlation between chronological age and DNAmAge in controls and IPF patients before (T0) and after treatment (T1), using different methods and epigenetic clocks. **A** DNAmAge estimates in healthy controls obtained using the Horvath (Pearson r = 0.954, *p* < 0.0001), Levine (r = 0.882, *p* = 0.0007), Horvath Skin & Blood (r = 0.993, *p* < 0.0001), Hannum (r = 0.980, p < 0.0001), BLUP (r = 0.990, *p* < 0.0001), and EN (r = 0.987, *p* < 0.0001) clocks with the Illumina MethylationEPIC array and HumanMethylation450 BeadChip, and the 5-CpG methylation panel (r = 0.974, p < 0.0001) plotted against chronological age. **B** DNAmAge estimates in IPF patients at baseline (T0) using the Horvath clock (Pearson r = 0.781, *p* < 0.0001), Levine clock (PhenoAge) (r = 0.728, *p* < 0.0001), 5-CpG panel DNAmAge (r = 0.895, p < 0.0001), Horvath Skin & Blood clock (r = 0.850, *p* < 0.0001), Hannum clock (r = 0.909, *p* < 0.0001), BLUP clock (r = 0.920, *p* < 0.0001), EN clock (r = 0.961, *p* < 0.0001). **C** DNAmAge estimates in IPF patients at follow-up (T1) using the Horvath clock (Pearson r = 0.756, *p* < 0.0001), Levine clock (PhenoAge) (r = 0.735, *p* < 0.0001), 5-CpG panel DNAmAge (r = 0.846, *p* < 0.0001), Horvath Skin & Blood clock (r = 0.905, *p* < 0.0001), Hannum clock (r = 0.892, *p* < 0.0001), BLUP clock (r = 0.904, *p* < 0.0001), EN clock (r = 0.948, *p* < 0.0001). Dotted lines represent identity lines corresponding to chronological age.

In healthy controls, a strong and significant positive correlation was observed between chronological age and DNAmAge across all methods (Fig. 2A), including Horvath (r = 0.954, *p* < 0.0001), Levine (r = 0.882, *p* = 0.0007), Skin & Blood (r = 0.993, *p* < 0.0001), Hannum (r = 0.980, *p* < 0.0001), BLUP (r = 0.990, *p* < 0.0001), EN (r = 0.987, *p* < 0.0001), and the 5-CpG panel (r = 0.974, *p* < 0.0001), confirming the robustness and consistency of DNAmAge estimation across platforms. A similar age-dependent relationship was observed in IPF patients both at baseline (T0) and follow-up (T1), with significant correlations across all clocks (Fig. 2B, C, all *p* < 0.0001). Correlation coefficients remained high across methods, with particularly strong associations for EN and BLUP clocks, indicating stable performance across timepoints.

Notably, DNAmAge estimates derived from the Skin & Blood clock consistently showed the greatest underestimation of chronological age across both controls and IPF patients, suggesting a systematic offset related to clock calibration rather than disease-specific effects.

When the predicted 5-CpG DNAmAge was used, the positive correlation with chronological age was also confirmed (Supplementary Fig. 1); however, the strength and dispersion of the association were comparable to those obtained with the absolute 5-CpG panel DNAmAge, indicating that the prediction model did not improve deviation from chronological age.

Based on the methodological comparison, the 5-CpG panel DNAmAge—requiring a lower amount of input DNA compared with array-based approaches—allowed DNAmAge and age acceleration analyses to be performed in a larger subset of IPF patients (*n* = 31) for whom sufficient DNA was available.

**Fig. 3:**
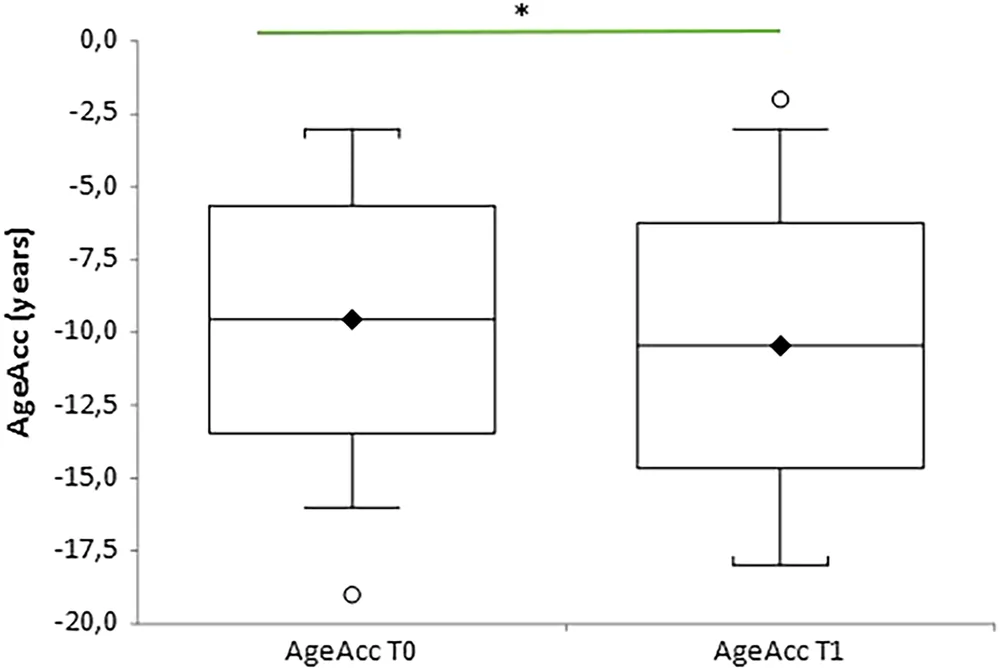
AgeAcc of blood leukocytes at T0 and T1 in the subgroup of IPF patients (*n* = 31). Box plots show levels of blood leukocytes AgeAcc at T0 and T1 in the subgroup of IPF patients (*n* = 31). In box plots, the boundary of the box closest to the x-axis indicates the 25th percentile, the line within the box marks the mean, and the boundary of the box farthest from the x-axis indicates the 75th percentile. Whiskers (error bars) above and below the box indicate the 95 and 5th percentiles. The horizontal bar with asterisk indicates the significant comparison between blood leukocytes AgeAcc at T0 and T1 (*Paired *t* test: mean AgeAcc (years) at T1, –10.45 ± 4.22 vs mean AgeAcc (years) at T0, –9.55 ± 3.91; *p* = 0.0435).

At follow-up (T1), a significant reduction in epigenetic age acceleration (AgeAcc) was observed compared with baseline (–10.45 ± 4.22 at T1 vs –9.55 ± 3.91 at T0; *p* = 0.0435; Fig. 3). In contrast, unadjusted DNAmAge values (i.e., not corrected for chronological age) did not differ between T1 and T0 (*p* > 0.9999).

Multiple linear regression analyses (Table 3 and Supplementary Table 2) showed that both 5-CpG panel DNAmAge and AgeAcc at T1 were strongly determined by their respective baseline values (*p* < 0.0001). Male sex was associated with higher DNAmAge (*p* = 0.0518) and significantly higher AgeAcc (*p* = 0.0123) at T1, while no other covariates reached statistical significance.

In longitudinal models assessing changes over time (Table 4 and Supplementary Table 3), the increase in AgeAcc (ΔAgeAcc = AgeAcc T1–T0) was significantly associated with male sex (*p* = 0.0204). No significant predictors were identified for longitudinal changes in DNAmAge (ΔDNAmAge).

**Fig. 4:**
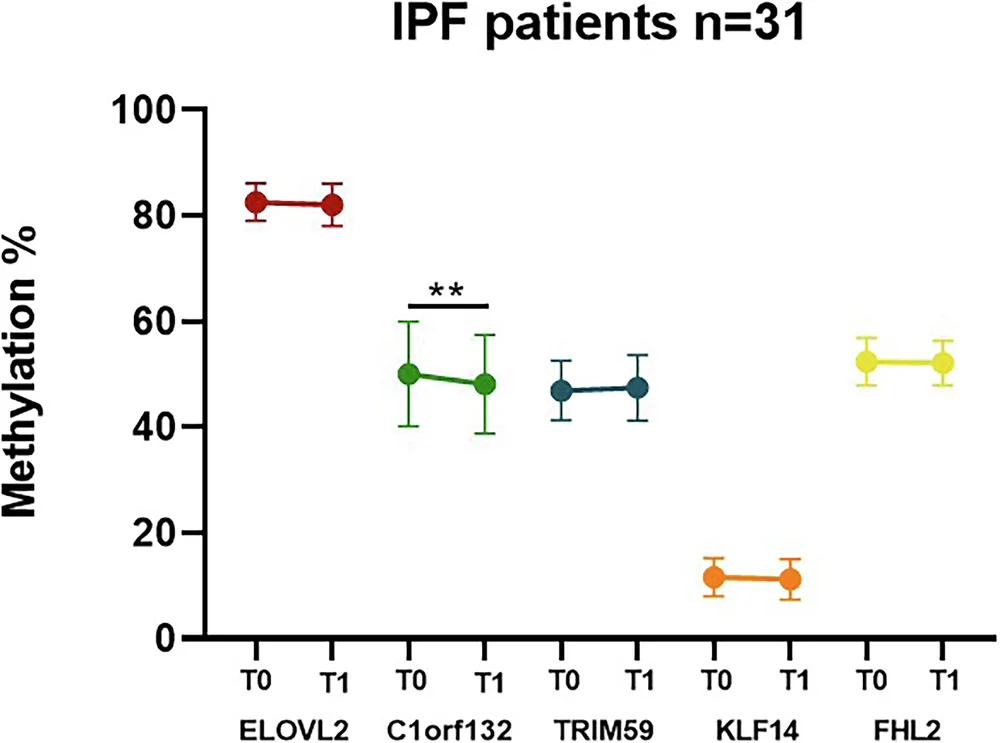
DNA methylation levels of the 5 CpG panel DNAmAge before and after treatment in IPF patients (*n* = 31). DNA methylation status at the CpG sites of the five genes (ELOVL2, C1orf132, KLF14, TRIM59, and FHL2) analyzed for the 5-CpG panel DNAmAge determination, reported as percentage of methylation (%), before (T0) and after (T1) treatment in *n* = 31 IPF patients. Data are reported as mean ± SD. Student’s *T* test was used for statistical analyses; **, *p* < 0.01.

Finally, CpG-specific methylation analysis of the five loci included in the 5-CpG DNAmAge panel (ELOVL2, C1orf132, KLF14, TRIM59, and FHL2) revealed a significant reduction in methylation at the C1orf132 locus following antifibrotic treatment (*p* = 0.0044; Fig. 4), whereas no significant changes were detected at the other CpG sites.

**Fig. 5:**
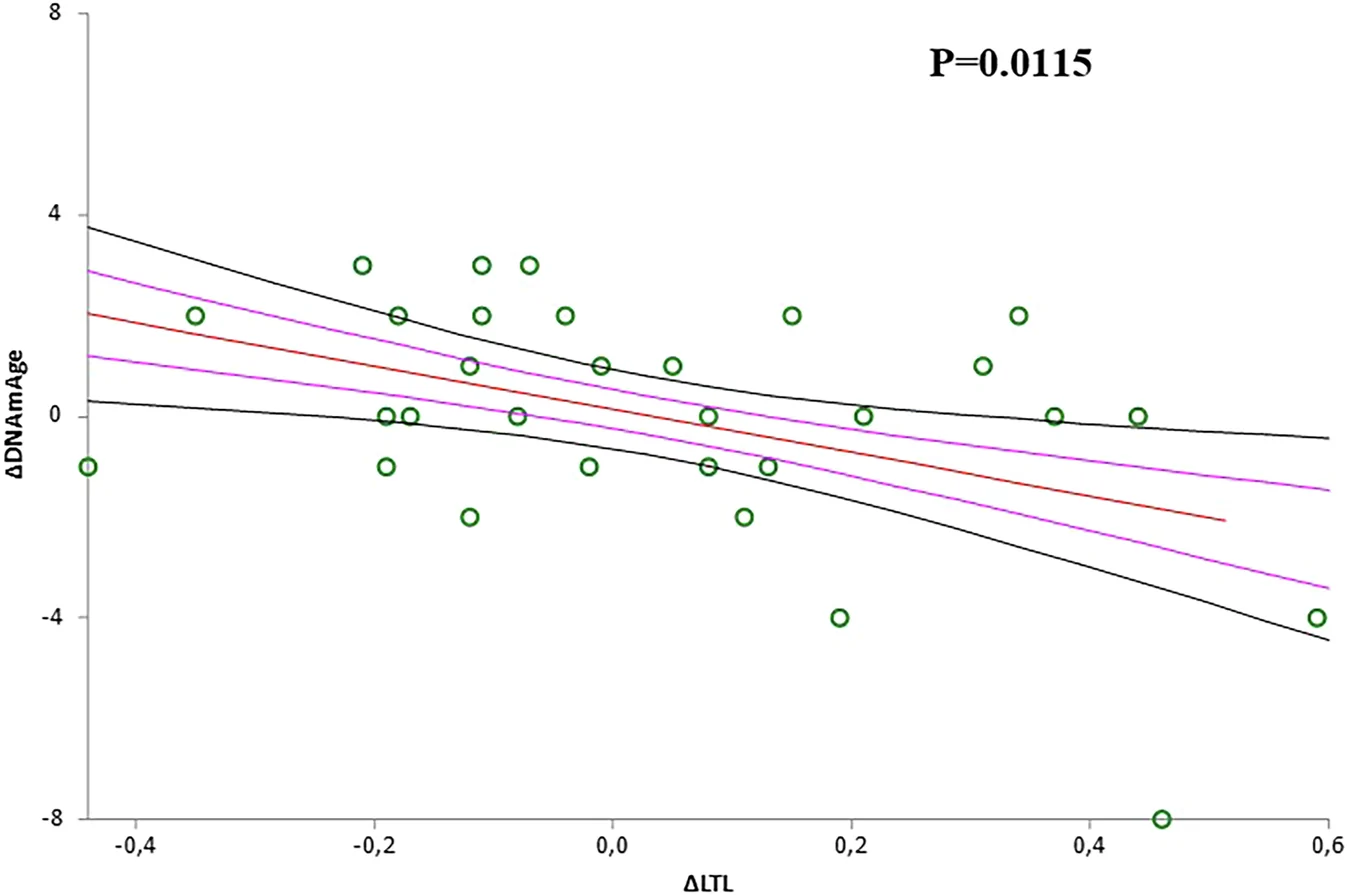
Correlation curve between Δ5-CpG panel DNAmAge and ΔLTL of blood leukocytes in the subgroup of IPF with follow up (*n* = 31). Simple linear regression plot shows the correlation between Δ5-CpG panel DNAmAge and ΔTL of blood leukocytes in the subgroup of IPF with follow up (*n* = 31) (Correlation coefficient (r) = –0.448). Mean, Standard Error (SE) and 95% coefficient intervals (CI) are represented as orange, pink and black lines, respectively.

The difference in sample size between analyses performed using the 5-CpG Qiagen panel (*n* = 31; Table 2) and those conducted using all three independent methodologies (*n* = 27; Figs. 1,2,5) is attributable to insufficient DNA availability to perform array-based DNAmAge analyses in four samples.

Simple linear regression analysis confirmed the negative correlation between the reduction in non-mitotic DNAmAge and the increase in LTL (ΔLTL = LTL T1-T0) (*p* = 0.0115), as illustrated in Fig. 5.

### Genotyping

Based on a literature review, 17 SNPs previously associated with IPF and included in the Infinium Global Screening Array (GSA) were selected for analysis. Supplementary Table 6 reports the rs and IDs of the 17 variants, the corresponding risk alleles (EA) according to the analyzed literature, and the respective references. The allelic frequencies (%) of the EA determined for each of the 17 SNPs in the 48 genotyped IPF patients are showed in Supplementary Table 7. In our cohort, EA frequencies ranged from a maximum of 84.3% for rs1981997 (*MAPT*) to a minimum of 1% for rs3893252 (*DAZAP1*). Among the most represented alleles, rs1981997 (*MAPT*, 84.3%) and rs5743890 (*TOLLIP*, 82.2%) showed high prevalence but were relatively comparable to reference datasets, whereas rs1278769 (*ATP11A*, 83.3%) demonstrated a clearly elevated frequency compared to both European and worldwide populations. Conversely, rs3893252 (*DAZAP1*) and rs4387287 (*OBFC1*) displayed markedly reduced frequencies in Italian IPF patients as shown in Supplementary Table 7.

**Fig. 6:**
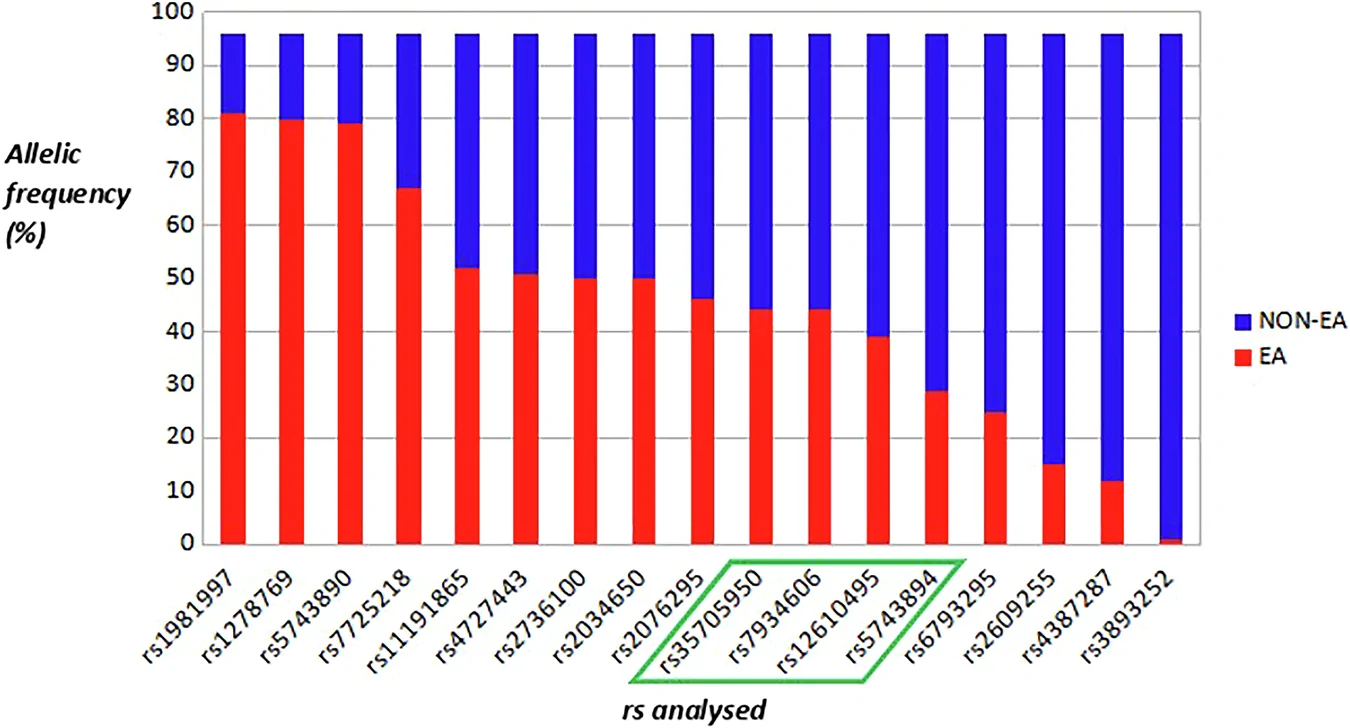
Allele frequency distribution of effect (EA) and non-effect (NON-EA) alleles in the 48 genotyped IPF patients. For each analyzed SNP (n = 17), allele frequencies (%) are shown, with EA displayed in red and NON-EA in blue. Variants remaining significant after chi-square testing with multiple-testing correction are boxed in green (see Table 5).

To formally test whether the observed allele frequencies differ from the EUR reference frequencies, we computed the chi-squared tests with the multiple-testing correction of Benjamini–Hochberg (Table 5). In four variants (Significant p_chi2_BH values are shown in bold in Table 5), the frequency of the EA in cases differs from the expected frequency in the reference population; therefore, the EA in cases is enriched or depleted compared with what is predicted based on European population frequencies derived from NCBI. The allelic distribution is presented in Fig. 6, with rs12610495 (*p* = 0.05), rs35705950 (*p* = 6.95 × 10⁻⁴⁵), rs5743894 (*p* = 0.00708), and rs7934606 *p* = 0.05 that passed the chi-squared test with multiple-testing correction.

**Table 5:** Chi-square tests comparing allele distributions between cases and a reference European population, with *p* values adjusted for multiple testing using the Benjamini–Hochberg correction

	p_ref	Freq E	Freq N	E_cases	N_cases	p_chi2	p_chi2_BH
rs11191865	0.491392	0.5417	0.4583	52	44	0.33	0.56
rs1278769	0.756553	0.8333	0.1667	80	16	0.08	0.24
**rs12610495**	**0.288395**	**0.4062**	**0.5938**	**39**	**57**	**0.01**	**0.05**
rs1981997	0.809882	0.8437	0.1563	81	15	0.40	0.58
rs2034650	0.519188	0.5208	0.4792	50	46	0.97	0.97
rs2076295	0.452016	0.4791	0.209	46	50	0.59	0.72
rs2609255	0.222035	0.1563	0.8437	15	81	0.12	0.30
rs2736100	0.493517	0.5208	0.4792	50	46	0.59	0.72
**rs35705950**	**0.07189**	**0.4583**	**0.5417**	**44**	**52**	**4.09E–46**	**6.95E–45**
rs4387287	0.17279	0.125	0.875	12	84	0.22	0.46
rs4727443	0.617254	0.5313	0.4687	51	45	0.08	0.24
rs5743890	0.853116	0.8229	0.1771	79	17	0.41	0.58
**rs5743894**	**0.172257**	**0.302**	**0.698**	**29**	**67**	**8.32E-4**	**7.08E-3**
rs6793295	0.265075	0.2604	0.7396	25	71	0.92	0.97
rs7725218	0.648503	0.6979	0.3021	67	29	0.31	0.56
**rs7934606**	**0.585276**	**0.4583**	**0.5417**	**44**	**52**	**0.01**	**0.05**
rs3893252	0.009232	0.0104	0.9896	1	95	0.90	0.97

RSID: reference sequence of each variant associated with IPF, p_ref: frequency of the effect allele in the European population (NCBI), Freq E: observed frequency of the effect allele in the 48 analyzed individuals, Freq N: observed frequency of the non-effect allele in the 48 analyzed individuals, E_cases: number of effect alleles within the analyzed population, N_cases: number of non-effect alleles within the analyzed population, p_chi2: chi-square *p* value; p_chi2_BH: BH-corrected chi-square *p* value (Benjamini–Hochberg).

p_chi2_BH in bold are significant: four variants that passed the chi-squared test with multiple-testing correction. These variants show a statistically significant difference in effect allele frequency in the analyzed population compared with the European reference population.

A synthetic reference population was generated based on expected effect allele frequencies to allow contingency table–based statistical testing.

In particular, two of the four IPF-associated variants identified in our cohort—rs12610495 (*p* = 0.05) and rs35705950 (*p* = 6.95 × 10⁻⁴⁵)—are also included in the polygenic risk score (PRS) for IPF proposed by Wang et al.^41^, developed in a European population (Table 6). Within that PRS, rs35705950 (MUC5B) is characterized by a high effect weight (1.5769) and an odds ratio of 4.84, while rs12610495 (DPP9) shows a positive effect weight (0.2700) and an odds ratio of 1.31.

**Table 6:** Significant variants according to the chi-square test with multiple-testing correction included in the Xiaojie Wang et al. work (DOI: 10.1016/j.chemosphere.2023.139362, PGS Catalog ID: PGS004695)

rsID	chr	position	effect_allele	other_allele	effect_weight	AF_effect	locus_name	OR
**rs12610495**	19	4717672	G	A	0.270027	0.305	DPP9	1.31
**rs35705950**	11	1241221	T	G	1.576915	0.149	MUC5B	4.84

RSID: reference sequence of each variant associated with IPF.

chr: chromosome.

position: base pairs position.

effect_allele: allele tested for the association with IPF.

other_allele: alternative allele.

effect_weight: numerical value that quantifies the magnitude and direction of the association between each variant and the disease of interest.

AF_effect: frequency of the effect allele reported in the dataset by Xiaojie Wang et al.^41^.

locus_name: locus name in which the variant is located.

OR: odds ratio (OR >1 the allele is associated with an increased risk of the disease, OR < 1 the allele is associated with a protective effect).

The cumulative distribution of EA per individual revealed considerable inter-individual variability within the IPF cohort. The number of EAs per subject ranged from 10 to 22, with a cohort mean of 16 EAs (Supplementary Fig. 2), underscoring the broad genetic heterogeneity among patients.

A multiple regression analysis (Table 7) was performed to assess the influence of age, BMI, gender, LTL at diagnosis, and total EAs on forced vital capacity (FVC). The results demonstrated that the total number of EAs was a significant determinant of decreased FVC (*p* = 0.0136), together with gender, with female patients showing a stronger association with reduced FVC (*p* = 0.0003).

**Table 7:** Multiple regression analysis: the influence of age (years), BMI (Kg/m2), gender (M = 1; F = 0), LTL at diagnosis, and total effect alleles (EAs) on Forced Vital Capacity (FVC)

	Variables	b	r	t	*P* value
**Forced vital Capacity (FVC)**.	Age (years)	–0.018713	–0.238295	–1.590134	0.1193
	BMI (Kg/m^2^)	–0.01139	–0.065749	–0.427024	0.6715
	**Gender (m** = **1; F** = **0)**	**0.917286**	**0.52177**	**3.963787**	**0.0003**
	**Total EAs**	**–0.085527**	**–0.369452**	**–2.576621**	**0.0136**
	LTL (T/S)	0.398756	0.210682	1.396729	0.1698

*BMI* body mass index, *FVC* forced vital capacity, *LTL* leukocyte telomere length.

Significant *P* values are shown in bold.

### Polygenic risk score analysis

To further contextualize this targeted genetic approach within a genome-wide framework, genotype imputation followed by polygenic risk scores (PRS) analysis was performed. To extend the genetic analysis beyond directly genotyped variants, genotype imputation was performed, yielding approximately 39 million variants after quality filtering (R² ≥0.8). This enabled the application of two previously validated PRS derived from European populations (see Supplementary Table 8 for details of PRS composition and imputation metrics).

**Fig. 7:**
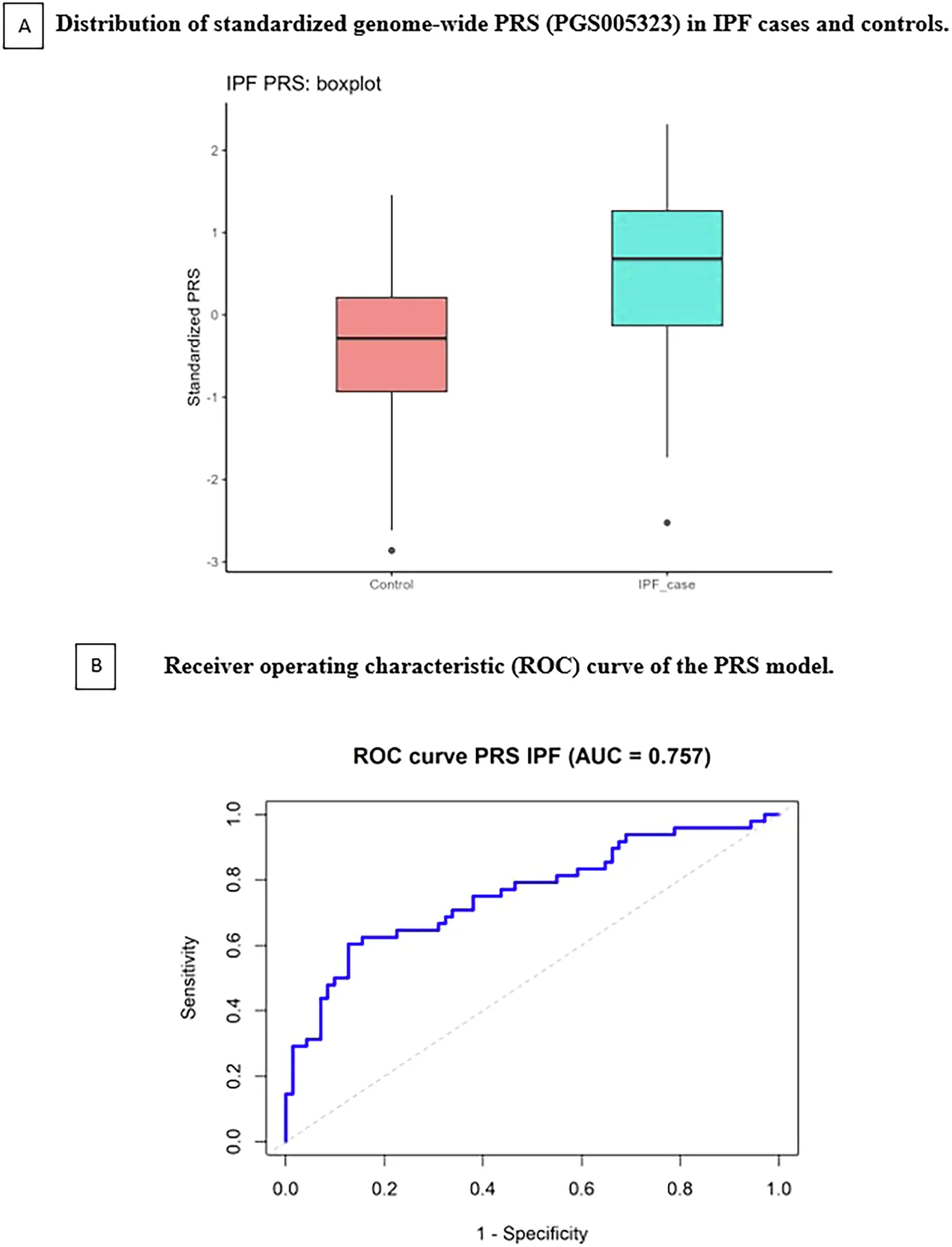
Polygenic risk score distribution and discriminative performance. Distribution of standardized genome-wide PRS (PGS005323) in IPF cases and controls. Receiver operating characteristic (ROC) curve of the PRS model. **A** Distribution of standardized genome-wide PRS (PGS005323) in IPF cases and controls. Graph shows significantly higher values in cases (mean cases: 0.51; mean controls: −0.34; *p* = 4.8 × 10⁻⁶). **B** Receiver operating characteristic (ROC) curve of the PRS model. The model demonstrated moderate discriminatory ability, with an area under the ROC curve (AUC) of 0.757.

For the genome-wide PRS (PGS005323), 605362 variants (92.6% of the original model) were retained after imputation filtering. PRS values were significantly higher in IPF patients compared to controls (mean cases: 0.51; mean controls: −0.34; *p* = 4.8 × 10⁻⁶), with a clear separation of distributions (Fig. 7A). Detailed performance metrics are reported in Supplementary Table 9. Logistic regression analysis showed that each one standard deviation increase in PRS was associated with a higher likelihood of IPF (OR = 2.88). The model demonstrated moderate discriminatory performance, with an area under the ROC curve (AUC) of 0.757 (Fig. 7B). The variance explained by the model (Nagelkerke R²) was reduced after correction to the liability scale, yielding an estimate of approximately 6–7%, consistent with the polygenic nature of the disease.

For the targeted PRS (PGS004695), based on 22 variants after quality filtering (see Supplementary Table 10), a consistent pattern was observed. PRS values were significantly elevated in cases compared to controls (Supplementary Fig. 3A), and logistic regression analysis indicated a strong association with disease status (OR ≈ 3.78). The model showed good discriminatory ability, with an AUC of approximately 0.79 (Supplementary Fig. 3B). After correction for disease prevalence, the variance explained was approximately 9–10%.

Across both PRS models, IPF patients exhibited a higher genetic burden compared to controls, with effect sizes and variance explained in line with expectations for complex multifactorial diseases. Despite the significant association, the relatively low positive predictive value after prevalence correction highlights that PRS is more suitable for risk stratification rather than as a standalone diagnostic tool.

## Discussion

In this study, we conducted a comprehensive, multidimensional investigation into IPF by integrating telomere biology, epigenetic age acceleration metrics, and genetic susceptibility profiling in a well-characterized Italian cohort. Our approach provides new insights into the molecular heterogeneity of IPF and its modulation by antifibrotic treatment over time.

Our cohort—predominantly elderly men (mean age ~69 years), as expected for IPF^42^—showed no baseline association between chronological age and LTL in nonparametric and multivariable analyses, likely reflecting the relatively narrow age range. Conventional covariates, including age, BMI, and smoking status, were not independent determinants of baseline LTL. Importantly, IPF patients exhibited significantly shorter LTL compared with age- and sex-matched controls from our laboratory (Supplementary Table 11), in line with landmark studies^15,23,43^. Together, these findings reinforce telomere attrition as a central biological hallmark of IPF^23,44^, implicating premature cellular senescence and defective alveolar repair mechanisms as key contributors to disease onset and progression^40^. Clinically, shorter baseline LTL was associated with reduced diffusing capacity for carbon monoxide (DLCO)^23^, supporting LTL as a biomarker of disease severity and early prognostic stratification^23^. Notably, this evidence is consistent with the recent 2025 ISHLT Consensus Statement on Short Telomere Syndrome and Lung Transplantation, which underscores the clinical relevance of telomere biology in interstitial lung disease progression and transplant-related risk assessment^45^.

At follow-up (T1), we performed a comprehensive longitudinal assessment of biological aging biomarkers—including LTL, DNAmAge, and AgeAcc—to investigate their associations with clinical outcomes, treatment exposure, and demographic or occupational factors. LTL remained generally stable or exhibited a slight, non-significant increase compared with baseline. However, multiple regression analyses revealed that LTL at T1 was significantly influenced by both treatment duration and baseline LTL values. Notably, patients receiving nintedanib showed a greater increase in LTL compared with those treated with pirfenidone, and this effect was positively correlated with treatment duration. These findings suggest that nintedanib may exert a protective effect on telomere maintenance, an intriguing hypothesis that warrants further mechanistic exploration. Nintedanib, a tyrosine kinase inhibitor, targets multiple growth factor receptors involved in fibrotic processes, including the platelet-derived growth factor receptor (PDGFR), fibroblast growth factor receptor (FGFR), and vascular endothelial growth factor receptor (VEGFR)^36^. Its antifibrotic activity is primarily mediated through inhibition of fibroblast proliferation, differentiation, and migration, as well as by reducing extracellular matrix (ECM) deposition. Moreover, nintedanib has been reported to attenuate vascular remodeling^46^. By dampening fibroblast activation and turnover, nintedanib may mitigate the excessive cellular stress and replicative demand typical of fibrotic tissues, thereby slowing telomere attrition and contributing to the preservation of telomere length in patients with IPF. Recent preclinical evidence further suggests that nintedanib may exert senolytic properties by promoting apoptosis of senescent fibroblasts through inhibition of the STAT3 pathway^47^. Taken together, these data raise the possibility that the protective effects of nintedanib on telomere dynamics may extend beyond lung fibroblasts to circulating leukocytes, where we measured LTL in the present study. In line with our findings, these observations support a broader role for nintedanib in modulating aging-related mechanisms beyond fibrosis control and highlight LTL as a potential biomarker not only of disease progression^43^ but also of treatment response^48^. Furthermore, emerging therapeutic strategies aimed at preserving telomere integrity—such as sex hormone–based telomerase activation^49^ or intercellular telomere transfer via extracellular vesicles^50^—underscore the relevance of LTL monitoring within the framework of personalized medicine for IPF. Collectively, our findings open new avenues for precision medicine and aging modulation in fibrotic lung disease.

In parallel in this study we compared two methodologies for epigenetic age estimation—a targeted pyrosequencing assay of five well-established CpG sites (ELOVL2, FHL2, KLF14, PENK, TRIM59)^51,52^ and the Illumina MethylationEPIC BeadChip interrogating over 850,000 CpGs—using six independent clocks: Horvath^25^ and Levine (PhenoAge)^53^ clock, Skin & Blood clock^54^, Hannum clock^26^, BLUP clock^55^, EN clock^55^. Across healthy controls and IPF patients, both before and after antifibrotic therapy, DNAmAge estimates were strongly correlated with chronological age for all methods, supporting the robustness and reproducibility of these approaches. Although DNAmAge estimates were strongly correlated with chronological age at baseline (T0), these correlations were consistently weaker at follow-up (T1), showing a similar pattern across all clocks. Even if not significant, this attenuation likely reflects increased variability in DNAmAge trajectories following antifibrotic treatment. Given the reduced sample size at follow-up, further studies are warranted to more thoroughly investigate this aspect. The Horvath^25^ and PhenoAge^53^ clocks were selected for their extensive validation and established clinical relevance. We acknowledge, however, that newer second- and third-generation epigenetic clocks may capture additional dimensions of biological aging. In this context, the Skin & Blood clock^54^, developed to improve age estimation in blood- and skin-derived samples, was also evaluated in our cohort. Notably, it did not provide substantial independent information, instead showing an even greater underestimation of DNAmAge. This finding further reinforces our results and argues against a clock-specific limitation, indicating that the observed offset is disease-related rather than methodological. From a translational perspective, while the Illumina EPIC array offers broader genomic coverage and high-resolution insights, it requires higher costs, greater DNA input, and complex bioinformatic workflows. In contrast, the 5-CpG pyrosequencing panel developed by Zbieć-Piekarska et al.^56^ and optimized by Pavanello et al.^51^ provides high feasibility, reproducibility, and minimal DNA requirements, allowing for rapid, cost-effective analyses suitable for routine clinical implementation. Accordingly, the targeted 5-CpG methylation panel was included as a complementary approach to genome-wide epigenetic clocks, offering practical advantages in terms of DNA input requirements and scalability. This approach is not intended to replace established epigenetic clocks such as Horvath or Levine, but rather to provide a complementary and scalable method for assessing age-related DNA methylation changes in clinical and longitudinal settings. This 5-CpG panel has been applied in multiple independent studies across epidemiological, clinical, and forensic contexts^51,52,57–67^, demonstrating robustness and sensitivity to biologically meaningful aging-related changes.

In the present study, we observed a significant reduction in epigenetic age acceleration (5-CpG panel AgeAcc) following antifibrotic therapy, while unadjusted DNAmAge remained unchanged. This pattern suggests that antifibrotic treatment may slow biological aging in IPF patients through epigenetic remodeling rather than direct resetting of the epigenetic clock. A plausible mechanism is the therapy-induced attenuation of chronic oxidative and inflammatory stress, well-established drivers of accelerated epigenetic aging^68^. Although absolute DNAmAge estimates showed a systematic offset in IPF patients, age acceleration metrics inherently account for such differences and remain informative for longitudinal analyses. Consistently, regression-based relative age acceleration analyses yielded concordant results, indicating that the observed changes are robust to the method used and reflect a disease-specific offset rather than a methodological artifact. Importantly, extending the analysis to additional epigenetic clocks (Hannum, BLUP, and EN) did not alter the overall interpretation of the results, as no consistent changes in age acceleration were observed after antifibrotic treatment across methods. This cross-clock concordance strengthens the robustness of our findings and indicates that the longitudinal epigenetic signal detected in this cohort is better captured by AgeAcc than by absolute DNAmAge estimates alone.

Notably, despite its simplified design, this targeted assay successfully captured biological aging acceleration in IPF, supporting its translational applicability. Interestingly, we also found that male patients exhibited higher 5-CpG panel DNAmAge and 5-CpG panel AgeAcc values at T1 compared with females, suggesting potential sex-specific trajectories in biological aging among individuals with IPF. This finding is consistent with previous evidence, including our own work in pauci- and asymptomatic COVID-19 healthcare workers^69^ and the study by Gallego-Fabrega et al.^70^ in patients with ischemic stroke, and Oblak et al.^29^, which together support the broader concept of the male-female health–survival paradox. According to this paradigm, males typically experience faster biological aging, despite often presenting lower disability rates compared with females^71^. Moreover, recent findings by Tondo et al.^72^ reinforce the relevance of sex differences in IPF, demonstrating that male sex significantly influences disease progression and long-term treatment response, with men showing poorer survival rates and reduced therapy tolerance compared with women. Taken together, these observations highlight the necessity of incorporating sex-specific considerations into clinical management and therapeutic decision-making for IPF. Furthermore, site-specific DNA methylation analysis of the five CpG loci used to compute the 5-CpG panel DNAmAge (ELOVL2, C1orf132, KLF14, TRIM59, FHL2) revealed a significant hypomethylation at the C1orf132 locus (chromosome 1 open reading frame 132) following antifibrotic treatment. C1orf132, also known as MIR29B2C, encodes a non-coding RNA located at 1q32.2 and is recognized as one of the most robust epigenetic predictors of chronological age. Consistent with previous studies^26,56,73^, methylation at C1orf132 typically declines with aging, and such hypomethylation is thought to enhance transcriptional activity, potentially modulating downstream regulatory pathways involving age-sensitive microRNAs. Interestingly, in specific pathological contexts—such as following hematopoietic stem cell transplantation (HSCT)—C1orf132 has instead been reported as hypermethylated, where it may influence graft function^74^. In our study, C1orf132 exhibited significant hypomethylation, confirming its strong correlation with age.

Finally, we observed a significant inverse correlation between ΔLTL and Δ5-CpG panel DNAmAge, supporting the existence of a shared biological aging axis that integrates telomeric and epigenetic mechanisms. Several investigations^75,76^, including our previous study^58^, have reported similar associations in blood-based measurements, further reinforcing the biological plausibility of this relationship. This convergence strengthens the potential utility of LTL and DNAmAge as complementary, integrative biomarkers for monitoring disease progression and assessing therapeutic efficacy in IPF.

To better understand the genetic underpinnings of IPF, we genotyped a subgroup of 48 patients for 17 SNPs previously identified in large genome-wide association studies (GWAS)^20,21,77–81^. This analysis revealed a complex genetic landscape characterized by selective allelic enrichment and marked inter-individual variability, consistent with the heterogeneous and polygenic nature of IPF susceptibility. Among the 17 variants analyzed, four SNPs—rs12610495 (DPP9), rs35705950 (MUC5B), rs5743894 (TOLLIP), and rs7934606 (MUC2)—remained significant after correction for multiple testing. Notably, two of these variants (rs35705950 and rs12610495) are also included in the polygenic risk score (PRS) for IPF proposed by Xiaojie Wang et al.^41^, developed in a European population. This overlap supports the relevance of these loci to genetic predisposition to IPF and provides external validation of our findings. Within this framework, rs35705950 (MUC5B) showed a very high effect weight ( ≈ 1.58) and an odds ratio close to 5, indicating that carriers of the T effect allele have an approximately fivefold increased risk of developing IPF compared with non-carriers. This represents one of the strongest single-variant effects reported for a complex disease such as IPF. In contrast, rs12610495 (DPP9) displayed a positive but more modest effect (OR = 1.31), consistent with the contribution of common variants of smaller effect size typically observed in polygenic disorders. Together, these results confirm the relevance of both variants in shaping IPF susceptibility.

The MUC5B promoter polymorphism rs35705950 emerged as the most prevalent and clinically significant variant in our cohort, in agreement with previous studies demonstrating its strong association with IPF in European populations^20,78,82^. MUC5B encodes a gel-forming mucin essential for mucociliary clearance (MCC) and respiratory host defense^83,84^. Carriers of the rs35705950 T allele exhibit markedly increased MUC5B expression in the airways, a feature that may initially enhance epithelial barrier function. However, excessive mucin production is thought to become maladaptive over time, impairing MCC efficiency, promoting mucus retention, and reducing the clearance of inhaled particles and pathogens. These processes may lead to repetitive epithelial injury and sustained activation of alveolar epithelial cells, thereby contributing to profibrotic remodeling^85^. Consistent with this view, dysregulated MUC5B expression has been associated with endoplasmic reticulum stress, persistent inflammation, epithelial cell death, and progressive extracellular matrix deposition^86–88^. Intriguingly, despite its strong association with disease susceptibility, the rs35705950 T allele has also been linked to improved survival in several cohorts^80,89–92^, suggesting a dual and context-dependent role in IPF pathogenesis. One possible explanation is that enhanced mucociliary defense may confer early protective effects, whereas chronic overexpression of MUC5B ultimately promotes fibrotic remodeling. This apparent paradox highlights the complex interplay between host defense mechanisms and fibrogenic pathways in IPF.

DPP9, which encodes a cytoplasmic serine protease involved in cell adhesion, migration, and apoptosis^93^, represents another established IPF risk locus. Although overall DPP9 expression appears only modestly altered in IPF lungs compared with controls, the rs12610495 variant has not been directly associated with changes in gene expression^20^. This observation suggests that its contribution to IPF susceptibility may involve indirect or context-dependent mechanisms affecting epithelial integrity, cell–cell interactions, or tissue remodeling, rather than simple transcriptional regulation.

Overall, the overlap between variants identified in our cohort and those included in an independently derived PRS supports the biological relevance of these loci in IPF susceptibility. At the same time, the limited sample size underscores the need for larger, well-powered studies to integrate genetic burden into comprehensive PRS frameworks and to better define how genetic susceptibility interacts with downstream biological aging processes and disease progression. While PRS represent the optimal framework for genetic risk assessment in IPF, technical constraints related to variant coverage limited their applicability in the present dataset. We therefore adopted an EAC based on well-established IPF susceptibility variants as a conservative and hypothesis-generating measure of cumulative genetic burden. Notably, the association between higher EAC and reduced lung function supports its biological relevance in this cohort. Nonetheless, genome-wide PRS analyses based on imputed data in larger, well-powered cohorts remain a key priority for future studies.

To further extend these findings from targeted genetic analyses to a genome-wide framework, we evaluated two previously validated and independent PRSderived from European populations. Both models consistently showed that IPF patients carry a significantly higher genetic burden compared with controls, supporting a measurable polygenic contribution to disease susceptibility. Notably, key variants identified in our targeted analysis, including loci in MUC5B, DPP9, TERT, and DSP, are represented within these PRS models, reinforcing the biological coherence of our findings. Despite moderate-to-good discriminative performance (AUC up to ~0.79), the variance explained by PRS after correction to the liability scale remained modest ( ~ 6–10%), consistent with the multifactorial nature of IPF. Furthermore, due to the low population prevalence of IPF, predictive values remain limited, indicating that PRS are not suitable as standalone diagnostic tools but may instead serve as complementary instruments for risk stratification, particularly when integrated with telomeric and epigenetic biomarkers. These findings support a model in which genetic susceptibility acts in concert with biological aging processes to shape disease risk and progression.

Recent advances in aging biology provide a unifying lens for our IPF findings. In particular, prior hallmark-based analyses^38^ have positioned IPF among the most aging-aligned diseases, with genetic susceptibility loci mapping to core aging-related pathways, including telomere maintenance and genomic stability (e.g., TERT, TERC, RTEL1, OBFC1), immune and senescence-associated signaling (e.g., DPP9, FAM13A, MUC5B), and deregulated nutrient sensing (e.g., DEPTOR). While we did not perform a formal hallmark decomposition analysis in the present study, we use this framework here for interpretative purposes, and the convergence of telomeric, epigenetic, and genetic signals observed in our cohort is consistent with this aging-aligned disease model and supports the rationale for biomarker-based stratification in IPF. In parallel, multiple ongoing phase II–III programs targeting aging-related mechanisms—such as senolytics, telomere-directed therapies, epigenetic modulators, and metabolic or kinase inhibitors—aim to slow disease progression and confer geroprotective effects. Within this context, our integrative framework—combining LTL, epigenetic age, and polygenic burden— may enable stratification by biological aging profile and support the future evaluation of hallmark-targeted interventions with measurable effects on biological age and clinical outcomes, positioning IPF at the interface of aging science and precision medicine.

This study has limitations—including sample size for patients in follow-up, single-country enrollment, follow-up duration, and platform heterogeneity—that warrant caution and independent validation. Prospective multicenter trials should embed LTL and DNAmAge endpoints to test whether biomarker improvements translate into slower functional decline and better survival, and mechanistic work should dissect telomere/epigenetic pathways. Such efforts will determine whether aging-informed, sex-aware, and genotype-guided strategies can operationalize precision medicine in IPF.

This study leverages a well-phenotyped Italian IPF cohort with longitudinal sampling, integrating telomeric, epigenetic, and genetic aging markers. Genetic analyses were exploratory and restricted to directly genotyped, well-established IPF susceptibility variants in a limited subset, prioritizing analytical robustness over genome-wide imputation. Within these constraints, we replicated key signals, including *MUC5B* and *DPP9*, and observed an association between cumulative genetic burden and reduced lung function. Longitudinal analyses revealed convergent dynamics between telomere length and epigenetic aging, reflected by the inverse relationship between ΔLTL and ΔDNAmAge, while the low-input 5-CpG methylation assay enabled scalable epigenetic profiling. Together, these features support the internal consistency and translational relevance of the study and underscore the need for future genome-wide, imputation-based analyses in larger IPF cohorts.

This work integrates telomere biology (LTL), epigenetic aging (DNAmAge/AgeAcc), and genetic susceptibility to provide a cohesive view of IPF as an aging-aligned disorder. At diagnosis, shorter LTL aligns with worse gas-exchange capacity, supporting LTL as a baseline severity marker. Longitudinally, antifibrotic therapy—particularly nintedanib—is associated with stabilization or modest gains in LTL and a reduction in AgeAcc, consistent with treatment-linked remodeling of aging pathways. The inverse coupling between longitudinal changes in LTL and DNAmAge further supports a shared biological aging axis integrating telomeric and epigenetic mechanisms. In parallel, enrichment of established IPF susceptibility variants, most notably MUC5B and DPP9, together with the association between cumulative effect-allele burden and reduced lung function, highlights the contribution of polygenic background to clinical heterogeneity. Collectively, these findings support a precision framework in which LTL, epigenetic clocks, and genotype are combined to refine risk, tailor surveillance, and track therapeutic benefit in IPF.

## Methods

### Study population

We conducted a longitudinal retrospective cohort study comprising *n* =101 patients affected by Idiopathic Pulmonary Fibrosis (IPF)—according to ATS/ERS/JRS/ALAT guidelines^94^ enrolled from July 2014 and followed- up in our center. The study was ethically approved by the *local Ethics Committee—University of Padova*, in accordance with principles of the Helsinki Declaration (practice number 3843/AO/16). All patients recruited, before starting treatment with antifibrotic drugs (nintedanib and pirfenidone), provided their written informed consent to participate to this study. Pulmonary Function Tests (PFTs) including Forced Vital Capacity (FVC% predicted) were recorded at diagnosis and during follow- up. These measurements were performed in accordance with the recommendations of the American Thoracic Society/European Respiratory Society^5,94^. At enrollment (T0), characteristics of the patients including demographic data (age, gender), lifetime history of smoking (pack-years) and body mass index (BMI), age at diagnosis and antifibrotic therapy were acquired through a questionnaire specifically structured. Blood samples were collected in vacutainers K3EDTA tubes and Paxgene tubes, for laboratory tests (total white blood cell counts) and leukocyte telomere length (LTL) determination.

After the first year of treatment a subgroup of 31 patients underwent a second blood analysis (T1) using the same methodology as at enrollment (T0). Data from these follow-up visits were integrated with occupational history, and biomarkers of biological aging, including LTL, DNA methylation age (DNAmAge), and Age Acceleration (AgeAcc), were analyzed.

Occupational history was systematically collected, and patients were considered as having an “occupational risk factor” (Table 2) if they reported at least ten years of exposure to agents documented in the literature as associated with IPF. Exposures of shorter duration or not previously linked to IPF were not included in this category.

**Fig. 8:**
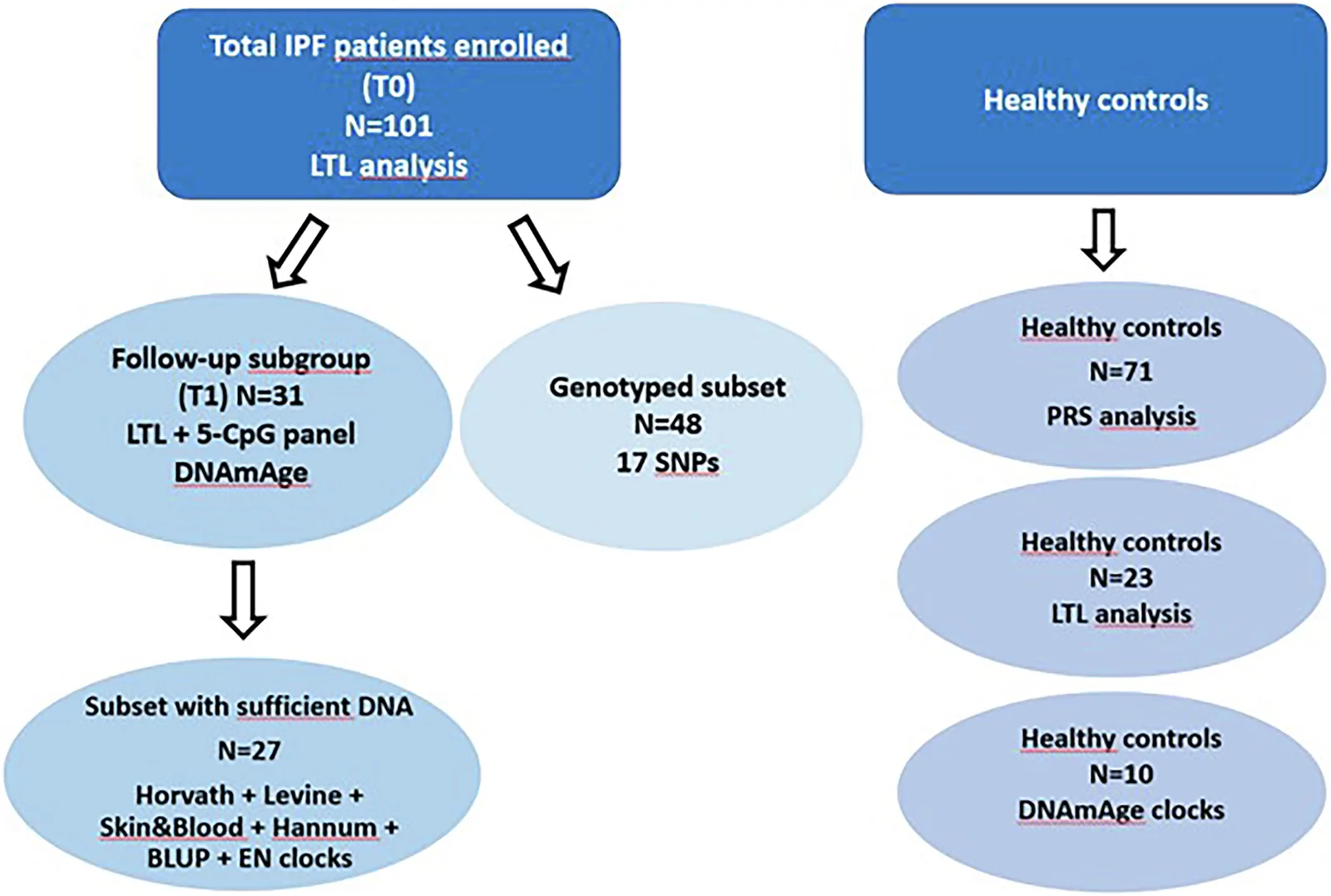
Study population and analytical workflow. Overview of the study population and analytical subgroups. A total of 101 patients diagnosed with idiopathic pulmonary fibrosis (IPF) were included at baseline (T0). Among them, 31 patients were re-evaluated after one year of antifibrotic treatment (T1) for longitudinal analyses. Genetic analyses were performed in a subset of 48 IPF patients genotyped for known IPF-associated variants. For polygenic risk score (PRS) analyses, an additional group of 71 healthy controls, free of known pulmonary disease and drawn from the same population, was included and genotyped using the same platform, enabling case–control comparison in a total dataset of 119 individuals. Epigenetic and telomere-based analyses were conducted in the available patients and controls subgroups as indicated.

Of the 101 IPF patients, a subset of *n* = 48 subjects were also genotyped to identify known genetic variants associated with the disease. For PRS-based analyses, an additional group of 71 healthy controls, free of known pulmonary disease and drawn from the same population, was included and genotyped using the same platform, enabling case–control comparison in a total dataset of 119 individuals. Fig. 8 provides an overview of the study population, detailing the number of IPF patients and controls enrolled and summarising the genetic and epigenetic assessments performed in each analytical subgroup.

### DNA extraction

DNA extraction was performed on all whole blood samples using an automated QIAcube System (QIAGEN, Milano, Italy) according to the DNAeasy Blood&Tissue kit (QIAGEN, Milano, Italy) as previously described^59^. After extraction, all DNA samples were quantified and checked for quality and integrity using QIAexpert Quantification System (Qiagen, Milano, Italy). We obtained genetic material suitable for subsequent analytical procedures from a quantitative (mean DNA 151.15 ng/µL) and qualitative (mean 260/280 = 1.84) point of view.

### Leukocyte telomere length (LTL) analysis

LTL in genomic DNA was measured by the real-time quantitative PCR method developed by Cawthon^95^ and previously described^60,96^. This method measures the relative LTL by determining the ratio of telomere repeat copy number (T) to single-copy gene (S) (T:S ratio) in experimental samples relative to the T/S ratio of a reference pooled sample^96^. The single copy gene employed in this investigation was the human β-globin (hbg). A “seven-points” standard curve was generated from a serially diluted DNA pool (obtained from DNA samples randomly selected) varying from 40 to 0.625 ng in each plate, in order to determine relative quantities of T and S (in nanograms). All samples and standards were analyzed in triplicate and the average of the 3 T/S ratio measurements was considered in the statistical analyses. A measure of T/S ratio was considered acceptable if the SD among triplicate measures was <0.25. To test the reproducibility of measurements, PCR runs of these samples were replicated in different days and the coefficient of variation (CV) for the average T/S ratio should be at least 10%^60,96^.

### DNAmAge analysis

DNAmAge was determined by using seven different epigenetic clocks such as the forensic method 5-CpG panel DNAmAge ^51,56^, the Horvath pan-tissue^25^, the Levine (PhenoAge)^53^ clock, the Skin & Blood clock^54^, and additional clocks (Hannum clock^26^, BLUP clock^55^, EN clock^55^) to provide a more comprehensive assessment of epigenetic aging.

According to the target approach method, DNAmAge was assessed by analyzing the methylation levels of five selected markers (ELOVL2, C1orf132, KLF14, TRIM59, and FHL2) in genomic DNA using bisulfite conversion and Pyrosequencing methodology as previously described^52,57^.

For genome-wide epigenetic clocks (Horvath, PhenoAge, Skin & Blood, Hannum, BLUP, and EN), DNAmAge was estimated based on methylation levels at predefined CpG sites (ranging from hundreds to thousands of loci depending on the model: 353^25^, 513^53^, 391^54^, 71^26^, 319607^55^, 514^55^ CpGs).

Sodium bisulfite conversion of genomic DNA was performed using the EZ DNA Methylation™ Kit according to the manufacturer’s instructions. Genome-wide DNA methylation was analyzed using the Illumina Infinium MethylationEPIC v2.0 BeadChip, following standard protocols. Arrays were scanned using the Illumina iScan SQ system to obtain probe intensity data. Raw IDAT files were processed and quality-controlled using a standardized pipeline implemented in R, including background correction, dye-bias normalization, and masking of low-quality CpGs. DNAmAge was then computed using established algorithms for each clock. GrimAge was not calculated due to incomplete CpG coverage in the dataset ( ~ 20% missing CpGs), which would have compromised the reliability of the estimates. Methylation levels were expressed as β-values (percentage of methylated cytosines at each CpG site) and used for DNAmAge estimation as previously reported^52,57^.

### Age acceleration (AgeAcc)

Age acceleration was defined as the difference between DNAmAge and chronological age (AgeAcc = DNAmAge – chronological age). The relative 5-CpG panel DNAmAge was also calculated by regressing 5-CpG panel DNAmAge on chronologic age and the relative 5-CpG panel AgeAcc by calculating the difference between the predicted relative 5-CpG panel DNAmAge and chronological age. As no substantial differences were observed between these approaches, absolute DNAmAge and AgeAcc values were used for subsequent analyses.

### Selection of IPF-associated SNPs

A literature-based SNP selection strategy was employed to identify genetic variants significantly associated with IPF susceptibility. The selection was informed by results from GWAS and meta-analyses published between 2008 and 2023, including the landmark study by Fingerlin et al.^20^ and more recent large-scale investigations^77–79^ that identified associations with telomere-related genes (*TERT*, *TERC*, *OBFC1*), mucosal defense (*MUC5B*), immune regulation (*DPP9*), and fibrosis pathways (*FAM13A*, *ATP11A*), as well as intergenic loci such as 7q22 and 15q14–15.

From this targeted review, a panel of 44 SNPs (Supplementary Table 12) was initially selected based on the following criteria:

- genome-wide significance (*p* < 5 × 10⁻⁸),
- strong biological plausibility and functional annotation,
- compatibility with the genotyping platform used.

This initial panel was cross-referenced with the content of the Illumina Infinium Global Screening Array (GSA) BeadChip, which interrogates over 650,000 genomic markers. Through this comparison, a final set of 17 SNPs present on the GSA array and suitable for genotyping was identified. These included SNPs in genes previously linked to IPF susceptibility and progression, enabling both replication of known findings and investigation of genotype–phenotype correlations.

### Genotyping and allelic frequency analysis

Genotyping was conducted on a selected subset of 48 patients diagnosed with IPF, specifically those for whom biological samples were available. The Infinium Global Screening Array (Illumina, USA) was used, following the manufacturer’s protocols. Rigorous quality control procedures were applied at both the sample and SNP levels to ensure high call rate accuracy and to minimize genotyping errors.

For each of the 17 selected SNPs, the effect allele (EA) frequency was determined in the study population and compared with corresponding reference frequencies derived from the dbSNP database (NCBI), focusing on global and European population data.

Additionally, the total number of EA per individual was calculated as a proxy of cumulative genetic burden. This metric was used to assess inter-individual variability and support exploratory analyses aimed at stratifying patients based on genetic risk profiles.

### Genotype imputation and polygenic risk score analysis

To extend the genetic analysis beyond directly genotyped variants, genotype imputation was performed on Global Screening Array (GSA) data. For PRS-based analyses, 48 IPF cases and an additional group of 71 healthy controls, free of known pulmonary disease and genotyped using the same platform, were included, resulting in a total dataset of 119 individuals for case–control comparison.

Prior to imputation, genotype coordinates were lifted over to the GRCh37 reference genome to ensure compatibility with the Haplotype Reference Consortium (HRC) reference panel implemented in the Michigan Imputation Server. Standard pre-imputation processing included allele harmonization, strand alignment, correction of strand flipping and allele swapping, and normalization of variant identifiers and genomic coordinates using the recommended Michigan Imputation Server pipeline. Variants located on chromosomes X, Y, MT, and XY were excluded prior to imputation. Imputation generated 22 chromosome-specific VCF files, which were subsequently merged into a single dataset comprising approximately 39 million variants. Variants with imputation quality R² < 0.8 were excluded from downstream analyses.

To robustly capture genome-wide genetic susceptibility, two previously validated and independent polygenic risk scores (PRS) derived from European populations were applied to the imputed dataset: PGS005323^97^ and PGS004695^41^. The genome-wide PRS (PGS005323) originally included 653482 variants; after quality filtering, 605362 variants (92.6%) were retained, and all 17 IPF-associated variants previously identified in our targeted analysis were represented in this model. The second PRS (PGS004695), based on a curated set of variants, originally included 23 loci; one low-frequency variant (rs537322302, HECTD2) was excluded due to low imputation quality (R² < 0.8), and the score was therefore computed using the remaining 22 variants.

PRS were calculated using PLINK2 (--score) and standardized as z-scores to enable interpretation per one standard deviation increase in genetic burden. Associations with IPF status were assessed using logistic regression models, from which odds ratios (ORs) were derived. Discriminatory performance was evaluated using receiver operating characteristic (ROC) curves and the corresponding area under the curve (AUC). The proportion of variance explained by each PRS was estimated using Nagelkerke’s pseudo-R² and subsequently transformed to the liability scale, assuming an IPF prevalence of 0.00015 in the European population^98^. Sensitivity, specificity, positive predictive value (PPV), and negative predictive value (NPV) were also calculated, with predictive values adjusted for disease prevalence. Differences in PRS distributions between cases and controls were assessed using Student’s *t* test or Wilcoxon test, as appropriate.

### Sample size estimation

For sample size estimation, we predicted a 10–15% decline in at least three biomarkers of biological age over the study period, with an expected standard deviation of 20%. To achieve sufficient power (two-sided alpha = 0.05/3, beta = 0.20, Bonferroni correction), we planned to enroll 25 subjects.

### Statistical analyses

Statistical analyses were performed on data based on information collected for each patient enrolled in the study. Continuous data were expressed as mean and standard deviation (SD), while categorical variables were given as percentages. Univariate and multivariate regressions were used selecting the appropriate models. The analyses were performed using the statistical software Stata and StastDirects and GraphPad Prism 8 software (GraphPad, USA). Comparisons between data were made by using (two-tailed) Paired t test for paired samples of the same patient (T0 and T1) in the subgroup patients (n = 31) underwent a follow up medical examination. Correlation was evaluated by a simple linear regression model in order to provide a measure of the strength of dependence between two variables. Multiple linear regression analysis was carried out at baseline on all *n* = 101 patients to assess the influence of independent variables – such as BMI, gender, pack-years, age, FVC, FVC%pred, blood count – on the dependent variable LTL.

Multiple linear regression analysis in a first model was also performed to evaluate the influence of the independent variables on the dependent variables LTL, 5-CpG panel DNAmAge, and 5-CpG panel AgeAcc at follow up (T1). A second model of multiple linear regression analysis was performed to assess their influence on ΔLTL, Δ5-CpG panel DNAmAge, and Δ5-CpG panel AgeAcc (T1-T0). Genetic risk profile data were integrated with clinical and molecular markers (e.g., LTL, DNAmAge) to explore potential associations with disease severity and progression.

Results were considered significant when a *p* value of ≤ 0.05 was obtained.

### Effect allele count (EAC)

To estimate the cumulative genetic predisposition to idiopathic pulmonary fibrosis (IPF), an effect allele count (EAC) was calculated for each genotyped individual. The EAC was constructed by summing the number of risk alleles (EA) carried by each subject across the 17 selected IPF-associated SNPs, identified through prior GWAS and meta-analyses. Each SNP was assumed to contribute additively to disease risk, and alleles were weighted equally, consistent with a simplified, unweighted EAC approach.

The score for each individual was computed as: EAC = Σ (number of effect alleles per SNP).

This score provides an approximation of individual genetic burden, allowing for stratification of patients based on their cumulative load of susceptibility variants. The distribution of EAC values across the cohort was analyzed to explore potential associations with clinical parameters, LTL, and epigenetic aging indices. Additionally, EAC values were compared to European population-level allele frequency data to contextualize the genetic risk observed in our Italian IPF cohort. Differences in allele frequencies were tested using chi-squared tests, with p-values adjusted for multiple testing using the Benjamini–Hochberg false discovery rate correction. In brief, for comparisons with the reference population, a *synthetic* reference population was generated based on the expected effect allele (EA) frequencies. Specifically, for each SNP, 10,000 alleles consistent with the reported EA frequency in the European reference population were simulated. This approach allowed theoretical allele frequencies to be converted into integer counts, enabling the application of contingency table–based statistical tests. For each SNP, the number of effect and non-effect alleles was compared between cases and the synthetic European reference population using chi-square tests. P-values were adjusted for multiple testing using the Benjamini–Hochberg correction.

## Supplementary information

Supplementary information

## Data Availability

Due to ethical and regulatory constraints related to informed consent and the sensitive nature of genetic and epigenetic data, individual-level genotype and DNA methylation data cannot be publicly deposited. The data that support the findings of this study are available from the corresponding author, SP, upon reasonable request and subject to approval by the local Ethics Committee. No high-throughput sequencing datasets or other publicly depositable raw omics datasets (e.g., RNA-seq, whole-genome sequencing, or proteomics) were generated in this study.
