# Supplementary material for: Telomere integrity, epigenetic aging, and genetic burden shape biological aging trajectories in idiopathic pulmonary fibrosis
Source: NPJ Aging. 2026 Jul 3;12(1):154. doi: 10.1038/s41514-026-00434-1 (PMC13754797; doi:10.1038/s41514-026-00434-1)
Supplement: Supplementary file 1 [file 41514_2026_434_MOESM1_ESM.pdf]

Supplementary information

Telomere Integrity, Epigenetic Aging, and Genetic Burden Shape Biological Aging Trajectories in Idiopathic Pulmonary Fibrosis

Manuela Campisi[1], Luana Cannella[2], Filippo Liviero[1,2], Federico Tamiazzo[3], Elisabetta Balestro[1,2], Paolo Spagnolo[1,2], Sofia Pavanello[1,2,4*].

[1] Department of Cardiac, Thoracic and Vascular Science and Public Health- DSCTV, University of Padua, Italy

[2] University - Hospital of Padova, Padova, Italy.

[3] BMR Genomics, via Redipuglia 21/A - 35131 Padova

[4] Centre of Studies and Activities for Space CISAS "G. Colombo" of University of Padua, Padua, Italy

* Corresponding author; email: sofia.pavanello@unipd.it

Supplementary Methods.

Genotype imputation and polygenic risk score analysis

For PRS-based analyses, 48 IPF cases and 71 healthy controls genotyped using the same Global Screening Array platform were included, resulting in a total dataset of 119 individuals. To enable imputation, genotype coordinates were lifted over to the GRCh37 reference genome and prepared using the standard Michigan Imputation Server scripts for allele harmonization, strand alignment, correction of strand flipping and allele swapping, and normalization of variant identifiers and genomic positions. Variants located on chromosomes X, Y, MT, and XY were excluded prior to imputation. Imputation was performed using the Michigan Imputation Server with the HRC reference panel and generated 22 chromosome-specific VCF files, which were subsequently merged into a single dataset comprising approximately 39 million imputed variants. Variants with imputation quality $R^2 < 0.8$ were excluded from downstream analyses.

Two previously published PRS derived from European populations were applied to the imputed dataset: PGS005323 and PGS004695. PGS005323 originally comprised 653,482 variants, of which 605,362 (92.6%) were retained after imputation quality filtering. All 17 IPF-associated variants previously selected in our targeted analysis were represented in this score. PGS004695 originally comprised 23 variants; one low-frequency variant (rs537322302, HECTD2) was excluded because of low imputation quality ($R^2 < 0.8$), and the score was therefore calculated using the remaining 22 variants.

PRS were calculated using PLINK2 (--score) and standardized as z-scores. Associations with disease status were evaluated using logistic regression, and discriminatory performance was assessed by ROC curves and area under the curve (AUC). Variance explained was estimated using Nagelkerke's pseudo-R^2 and transformed to the liability scale assuming an IPF prevalence of 0.00015 in the European population. Sensitivity, specificity, PPV, and NPV were also estimated, with predictive values corrected for disease prevalence. Differences in PRS distribution between cases and controls were assessed using Student's t-test or Wilcoxon test, as appropriate.

Supplementary Tables

Supplementary Table 1. Multiple regression analysis: the influence of independent variables at T0 as BMI (Kg/m^2), gender, pack-years ((cigarettes/20)xyears), age, FVC, FVC percent of the predicted value (FVC%pred), leucocytes (x10^3/ml), neutrophils (x10^3/ml), lymphocytes (x10^3/ml) and monocytes (x10^3/ml), on the dependent variable LTL.

Variables	B	R	t	p
BMI	b1=-0.001	r=-0.016	t=-0.140	P=0.889
Gender	b2=-0.063	r=-0.040	t=-0.355	P=0.723
Pack Years	b3=0.001	r=0.067	t=0.597	P=0.552
Age	b4=-0.005	r=-0.106	t=-0.945	P=0.347
FVC	b5=0.066	r=0.063	t=0.559	P=0.577
FVC%pred	b6=0.001	r=0.021	t=0.185	P=0.854
Leucocytes (x10^3/ml)	b7=0.0003	r=0.099	t=0.881	P=0.381
Neutrophils (x10^3/ml)	b8=0.034	r=0.132	t=1.181	P=0.241
Lymphocytes (x10^3/ml)	b9=-0.040	r=-0.081	t=-0.721	P=0.473
Monocytes (x10^3/ml)	b10=-0.077	r=-0.040	t=-0.357	P=0.722

Acronyms: BMI=body mass index; **FVC**= forced vital capacity**; FVC(%pred)**= forced vital capacity (percentage of the predicted normal value).

Supplementary Table 2. Multiple regression analysis: the influence of BMI (Kg/m2), gender (M=1; F=0), pack-years ((cigarettes/20) x years), lung function decline (FVC%pred T1-T0), age at beginning of follow-up T0 (years), LTL / 5-CpG panel DNAmAge / 5-CpG panel AgeAcc at (T0), duration of treatment (days), and occupational risk factor on LTL, 5-CpG panel DNAmAge and 5-CpG panel AgeAcc at follow up (T1).

	Variables	b	r	t	P-Value
T1 LTL	BMI	b1 = -0.006804	r = -0.125815	t = -0.59485	P = 0.558
	Gender (M=1)	b2 = 0.079917	r = 0.15917	t = 0.756214	P = 0.4575
	Pack-years	b3 = 0.000227	r = 0.023696	t = 0.111173	P = 0.9125
	Decline (FVC %pred T1-T0)	b4 = -0.004313	r = -0.150604	t = -0.714545	P = 0.4824
	Age T0	b5 = 0.003384	r = 0.110268	t = 0.520375	P = 0.608
	**LTL T0**	**b6 = 0.642611**	**r = 0.66351**	**t = 4.159686**	**P = 0.0004**
	**Duration of treatment (days)**	**b7 = 0.001157**	**r = 0.547459**	**t = 3.068487**	**P = 0.0056**
	Occupation	b8 = 0.209806	r = 0.367963	t = 1.856121	P = 0.0769
	**Variables**	**b**	**r**	**t**	**P-Value**
T1 5-CpG panel DNAmAge	BMI	b1 = 0.153467	r = 0.292132	t = 1.432718	P = 0.166
	**Gender (M=1)**	**b2 = 2.083691**	**r = 0.401535**	**t = 2.056428**	**P = 0.0518**
	Pack-years	b3 = 0.004116	r = 0.04564	t = 0.214294	P = 0.8323
	Decline (FVC %pred T1-T0)	b4 = -0.003256	r = -0.011911	t = -0.055871	P = 0.9559
	Age T0	b5 = 0.06019	r = 0.124768	t = 0.589824	P = 0.5613
	***5-CpG panel* DNAmAge T0**	**b6 = 0.791651**	**r = 0.860308**	**t = 7.915657**	**P < 0.0001**
	Duration of treatment (days)	b7 = -0.003525	r = -0.203398	t = -0.974389	P = 0.3405
	Occupation	b8 = -1.663566	r = -0.310627	t = -1.532792	P = 0.1396
	**Variables**	**b**	**r**	**t**	**P-Value**
T1 5-CpG panel AgeAcc*	BMI	b1 = 0.184112	r = 0.324503	t = 1.645299	P = 0.1135
	**Gender (M=1)**	**b2 = 2.826082**	**r = 0.493077**	**t = 2.718107**	**P = 0.0123**
	Pack-years	b3 = 0.000397	r = 0.004113	t = 0.019727	P = 0.9844
	Decline (FVC %pred T1-T0)	b4 = 0.052576	r = 0.191305	t = 0.93473	P = 0.3596

	5-CpG panel **AgeAcc T0**	**b5 = 0.861377**	**r = 0.871537**	**t = 8.524344**	**P < 0.0001**
	Duration of treatment (days)	b6 = -0.004858	r = -0.259676	t = -1.2896	P = 0.21
	Occupation	b7 = -0.602101	r = -0.123908	t = -0.598855	P = 0.5551

*The variable Age is not considered for AgeAcc because of its own definition.

Bold character is displayed only for significant values.

Acronyms: BMI=body mass index; **FVC(%pred)**= forced vital capacity (percentage of the predicted normal value); **LTL**=leukocyte telomere length; **DNAmAge**=DNA methylation age; **AgeAcc**=Age acceleration.

Supplementary Table 3. Multiple regression analysis: the influence of BMI (Kg/m^2), gender (M=1; F=0), antifibrotic therapy (0=pirfenidone; 1=nintedanib), pack-years ((cigarettes/20)×years), age at T0 (years), lung function decline (FVC%pred T1-T0), and duration of treatment (days) on *Δ*LTL (LTL T1-T0), Δ 5-CpG panel DNAmAge (DNAmAge T1-T0), Δ5CpG panel AgeAcc (AgeAcc T1-T0).

	Variables	b	r	t	P-Value
ΔLTL	BMI	b1 = -0.017549	r = -0.301378	t = -1.515836	P = 0.1432
	Gender (M=1)	b2 = 0.026736	r = 0.050506	t = 0.242528	P = 0.8105
	**Therapy (P=0; N=1)**	**b3 = 0.197156**	**r = 0.413044**	**t = 2.175101**	**P = 0.0402**
	Pack-years	b4 = -0.000796	r = -0.070951	t = -0.341129	P = 0.7361
	Age T0	b5 = -0.003064	r = -0.106249	t = -0.512451	P = 0.6132
	Decline (FVC %pred T1-T0)	b6 = -0.011056	r = -0.347259	t = -1.775912	P = 0.089
	**Duration of treatment (days)**	**b7 = 0.00093**	**r = 0.451936**	**t = 2.429691**	**P = 0.0233**
Δ5-CpG panel DNAmAge	**Variables**	**b**	**r**	**t**	**P-Value**
	BMI	b1 = 0.188671	r = 0.319576	t = 1.617452	P = 0.1194
	Gender (M=1)	b2 = 2.125313	r = 0.370654	t = 1.913919	P = 0.0682
	Therapy (P=0; N=1)	b3 = 0.450862	r = 0.102271	t = 0.493061	P = 0.6266
	Pack-years	b4 = -0.006255	r = -0.055343	t = -0.265824	P = 0.7927
	Age T0	b5 = -0.067492	r = -0.22748	t = -1.120328	P = 0.2741
	Decline (FVC %pred T1-T0)	b6 = 0.018415	r = 0.060917	t = 0.292693	P = 0.7724
	Duration of treatment (days)	b7 = -0.003082	r = -0.164296	t = -0.798792	P = 0.4326
Δ5-CpG panel AgeAcc*	**Variables**	**b**	**r**	**t**	**P-Value**
	BMI	b1 = 0.193313	r = 0.326992	t = 1.69511	P = 0.103
	**Gender (M=1)**	**b2 = 2.621248**	**r = 0.45209**	**t = 2.483017**	**P = 0.0204**
	Therapy (P=0; N=1)	b3 = 0.411273	r = 0.093707	t = 0.461098	P = 0.6489
	Pack-years	b4 = -0.006248	r = -0.055237	t = -0.27102	P = 0.7887
	Decline (FVC %pred T1-T0)	b5 = 0.043846	r = 0.152086	t = 0.753836	P = 0.4583
	Duration of treatment (days)	b6 = -0.005322	r = -0.277333	t = -1.414121	P = 0.1702

*The variable Age is not considered for AgeAcc because of its own definition.

Bold character is displayed only for significant values.

Acronyms: BMI=body mass index; **FVC(%pred)**= forced vital capacity (percentage of the predicted normal value); **LTL**=leukocyte telomere length; **DNAmAge**=DNA methylation age; **AgeAcc**=Age acceleration.

Supplementary Table 4 – DNAmAge (T0 vs T1 across clocks)

DNAmAge in IPF patients before (T0) and after (T1) antifibrotic treatment, estimated using a 5-CpG panel Qiagen system, and Illumina MethylationEPIC and 450K arrays (Horvath, Levine, Horvath Skin & Blood, Hannum, BLUP and EN clocks), and p-values comparing pre- and post-treatment measurements.

	Mean ± SD	Paired t-test
**Chronological age T0**	69.7 ± 7.54	**<0.0001**
**Chronological age T1**	70.6 ± 7.43	
**Horvath clock DNAmAge T0**	71.4± 7.20	0.653
**Horvath clock DNAmAge T1**	71.7± 6.69	
**Levine clock DNAmAge T0**	65.3± 9.69	0.356
**Levine clock DNAmAge T1**	66.1± 9.49	
**5-CpG panel DNAmAge T0**	60.6± 7.39	0.593
**5-CpG panel DNAmAge T1**	60.7± 6.81	
**Skin and blood clock DNAmAge T0**	54.5± 6.88	0.136
**Skin and blood clock DNAmAge T1**	55.2± 6.87	
**Hannum clock DNAmAge T0**	38.0± 7.19	**0.005**
**Hannum clock DNAmAge T1**	39.3± 7.00	
**BLUP clock DNAmAge T0**	67.3± 6.88	**0.015**
**BLUP clock DNAmAge T1**	67.9± 7.28	
**EN clock DNAmAge T0**	65.1± 7.71	**0.003**
**EN clock DNAmAge T1**	66.0± 7.89	

Acronyms: DNAmAge=DNA methylation age.

Supplementary Table 5 – AgeAcc (T0 vs T1 across clocks)

Age acceleration AgeAcc in IPF patients before (T0) and after (T1) antifibrotic treatment, estimated as difference between DNAmAge (calculated using a 5-CpG panel system and the Illumina MethylationEPIC and 450K arrays—Horvath, Levine, Horvath Skin & Blood, Hannum, BLUP, and EN) and chronological age, and p-values comparing pre- and post-treatment measurements.

	**Mean ± SD**	**P value ***
**Horvath clock AgeAcc T0**	1.65±4.89	0.285
**Horvath clock AgeAcc T1**	1.03± 4.98	
**Levine clock AgeAcc T0**	-4.41± 6.67	0.917
**Levine clock AgeAcc T1**	-4.503± 6.45	
**5-CpG panel AgeAcc T0**	-9.18± 3.42	0.057
**5-CpG panel AgeAcc T1**	-9.89± 4.00	
**Skin and blood AgeAcc T0**	-15.21± 3.01	0.563
**Skin and blood clock AgeAcc T1**	-15.46± 2.68	
**Hannum clock AgeAcc T0**	-31.78± 3.15	0.331
**Hannum clock AgeAcc T1**	-31.35± 3.38	
**BLUP clock AgeAcc T0**	-2.41± 2.96	0.265
**BLUP clock AgeAcc T1**	-2.69± 3.22	
**EN clock AgeAcc T0**	-4.64± 2.14	0.968
**EN clock AgeAcc T1**	-4.63± 2.51	

Acronyms: AgeAcc=Age acceleration.

Supplementary Table 6. Identification and selection by literature analysis of n=17 genetic variants included in the GSA associated with IPF disease. The following were identified in the GSA and were further selected. The table shows the rs ID of the n=17 rs identified, the risk allele according to the analyzed literature, as well as the non-effect allele, and the literature reference.

rsID	Risk allele	Non-effect allele	Reference
rs11191865	A	G	1
rs12610495	G	A	1
rs1278769	G	A	1
rs1981997	G	A	1
rs2034650	A	G	1
rs2076295	G	T	1
rs2609255	G	T	1
rs2736100	A	C	1
rs35705950	T	G	7
rs4387287	A	C	4
rs4727443	C	A	1
rs5743890	T	C	1
rs5743894	C	T	1
rs6793295	C	T	1
rs7725218	G	A	2
rs7934606	C	T	1
rs3893252	T	C	10

Supplementary Table 7. Comparison of allele effect frequencies in 48 Italian IPF patients with those in the worldwide and European populations.

rsID	Gene	Risk allele	Effect allele frequency (Worldwide)	Effect allele frequency (EUR)	Effect allele frequency (48 Italian IPF)
rs11191865	OBFC1	A	0.4716	0.4989	0.5417
rs12610495	DPP9	G	0.2705	0.2837	0.4062
rs1278769	ATP11A	G	0.7569	0.7567	0.8333
rs1981997	MAPT	G	0.8049	0.7865	0.8437
rs2034650	_ / Intronic	A	0.5105	0.5141	0.5208
rs2076295	DSP	G	0.4480	0.4494	0.4791
rs2609255	FAM13A	G	0.2350	0.2237	0.1563
rs2736100	TERT	A	0.4938	0.4903	0.5208
rs35705950	MUC5B	T	0.0264	0.0352	0.4583
rs4387287	OBFC1	A	0.1890	0.1749	0.1250
rs4727443	Intergenic	C	0.6096	0.6045	0.5313
rs5743890	TOLLIP	T	0.8634	0.8523	0.8229
rs5743894	TOLLIP	C	0.0935	0.1211	0.3020
rs6793295	LRRC34	C	0.2810	0.2615	0.2604
rs7725218	TERT	G	0.6388	0.6422	0.6979
rs7934606	MUC2	C	0.6371	0.5975	0.4583
rs3893252	DAZAP1	T	0.0452	0.0095	0.0104

Supplementary Table 8. Summary of genotype imputation and polygenic risk score models used in the study.

Parameter	PGS005323 (Zhu et al.)	PGS004695 (Wang et al.)
Population of derivation	European	European
Total variants in original PRS	653,482	23
Variants retained after QC ($R^2 \geq 0.8$)	605,362	22
Percentage of variants retained	92.6%	95.7%
Previously selected IPF SNPs included (n=17)	17/17	4/17
Variant excluded (if applicable)	–	rs537322302 (HECTD2)
Imputation reference panel	HRC	HRC
Imputation platform	Michigan Imputation Server	Michigan Imputation Server
PRS computation tool	PLINK2 (--score)	PLINK2 (--score)
Standardization	Z-score	Z-score

Supplementary Table 9. Performance metrics of the two polygenic risk scores in the case–control dataset (48 IPF cases and 71 controls).

Metric	PGS005323	PGS004695
Cases (n)	48	48
Controls (n)	71	71
Mean PRS (cases)	0.51	Higher than controls
Mean PRS (controls)	−0.34	Lower than cases
t-test p-value	4.8×10^{-6}	Significant
Wilcoxon p-value	2.1×10^{-6}	Significant
Odds Ratio (per 1 SD increase)	2.88	3.78
AUC (ROC analysis)	0.757	~0.79
Nagelkerke R^2	0.239	~0.34
Liability-scale R^2	~0.067	~0.095
Sensitivity	60.4%	~90%
Specificity	87.3%	~62%
PPV (corrected for prevalence)	0.000715	~0.0003
NPV (corrected for prevalence)	0.99993	Very high

Supplementary Table 10. List of genetic variants included in the PGS004695 polygenic risk score after imputation quality control. Variants previously identified in this study (Supplementary Table 4) are indicated. One variant (rs537322302, HECTD2) was excluded due to low imputation quality ($R^2 < 0.8$).

rsID	Gene / Locus	Previously identified in this study
rs878471	MCL1	No
rs2609279	FAM13A	No
rs2869966	FAM13A	No
**rs2076295**	**DSP**	**Yes**
rs2261360	ZKSCAN1	No
rs11191841	OBFC1	No
rs2304645	IVD	No
rs79412431	MAPT-AS1	No
rs78238620	KIF15	No
rs12696304	LRRC34 / TERC	No
rs2013701	FAM13A	No
**rs7725218**	**TERT**	**Yes**
rs12699415	MAD1L1	No
rs2897075	7q22.1	No
rs28513081	DEPTOR	No
**rs35705950**	**MUC5B**	**Yes**
rs9577395	ATP11A	No
rs59424629	IVD	No
rs62023891	AKAP13	No
rs2077551	MAPT	No
**rs12610495**	**DPP9**	**Yes**
rs41308092	RTEL1	No

Supplementary Table 11. Baseline characteristics of IPF patients and healthy controls.

	**IPF Patients**	**Controls**
**Number of subjects**	101	23
**Chronological age, years** *Mean ± SD*	69.0 ± 9.00	70.0 ± 11.5
**Sex (Male), n (%)**	84 (83%)	12 (52%)
**LTL (T/S ratio, 2018)** *Mean ± SD*	1.27 ± 0.34*	1.61 ± 0.61*

* Mann Whitney Test p=0.0177

Supplementary table 12. Literature-based panel of 44 SNPs associated with IPF susceptibility.

SNP	Gene [b]	Locus	Annotation	Reference
rs2736100	*TERT*	5p15		1
rs7725218	*TERT*	5		2
rs4727443		7q22	Intergenic	1
rs7934606	*MUC2*	11p15		2
rs2034650	*IVD*	15q14-15		1
rs1981997	*MAPT*	17q21		1
rs12610495	*DPP9*	19p13		1
rs6793295	*LRRC34*	3q26		1
rs12696304	*LRRC34 /TERC*	3		3
rs9860874	*TERC*	3		2
rs2609255	*FAM13A*	4q22		1
rs2609259	*FAM13A*	4		2
rs11191865	*OBFC1*	10q24		1
rs4387287	*OBFC1*	10		4
rs1278769	*ATP11A*	13q34		1
rs9577395	*ATP11A*	13		2
rs78238620	*KIF15*	3		5
rs2292181	*KIF15*	3		2
rs12699415	*MAD1L1*	7		6
rs12537430	*MAD1L1*	7		2
rs28513081	*DEPTOR*	8		5
rs10808505	*DEPTOR*	8		2
rs537322302	*HECTD2*	10		5
rs41308092	*RTEL1*	20		2
rs2076295	*DSP*	6		2
rs2897075	*ZKSCAN1*	7		2
rs79684490		10q25.1	Intergenic	2
rs35705950	*MUC5B*	11p15.5		7
rs2304645	*IVD*	15		2
rs12912339	*KNL1*	15		2
rs11073517	*AKAP13*	15		2
rs62025270	*AKAP13/KLHL25*	15		8
rs74614704	*NPRL3*	16		2
rs2077551	*MAPT*	17		2
rs112087793	*STMN3*	20		2
rs111521887	*TOLLIP*	11p15.5		9
rs5743894	*TOLLIP*	11p15.5		2
rs5743890	*TOLLIP*	11p15.5		2

rs7144383	*MDGA2*	14q21.3		9
rs17690703	*SPPL2C*	17q21.31		9
rs184498750	*SUCLG1*	2		10
rs60514164	*UBE2Q2*	15		10
rs35647788	*PCSK6*	15		10
rs3893252	*DAZAP1*	19		10

[b] Name of gene if SNP falls in body of gene

Supplementary Figures

Supplementary Figure 1. Correlation between chronological age and DNAmAge in IPF patients before (T0) and after treatment (T1), using different clocks, epigenetic and predicted epigenetic clocks.

A)

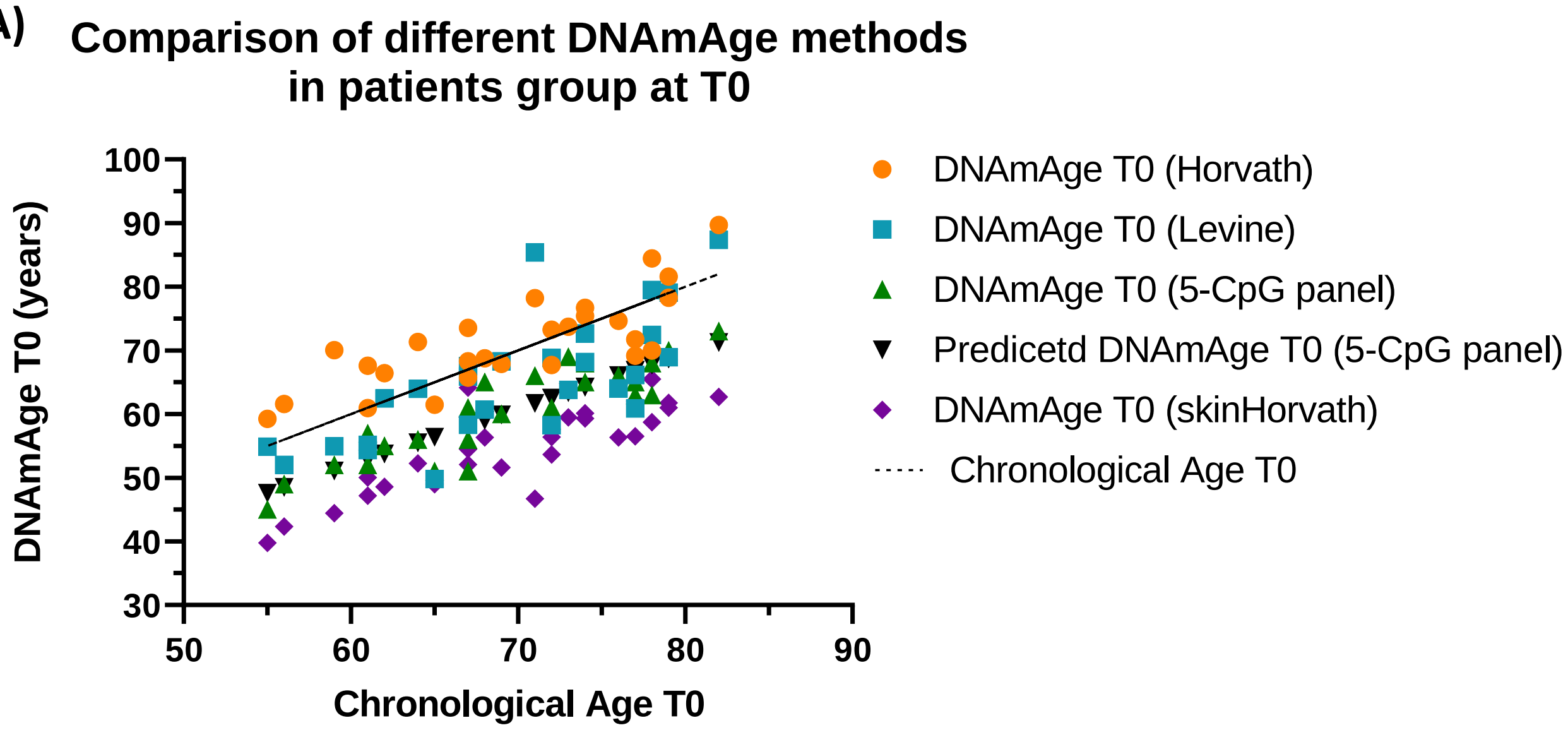

B)

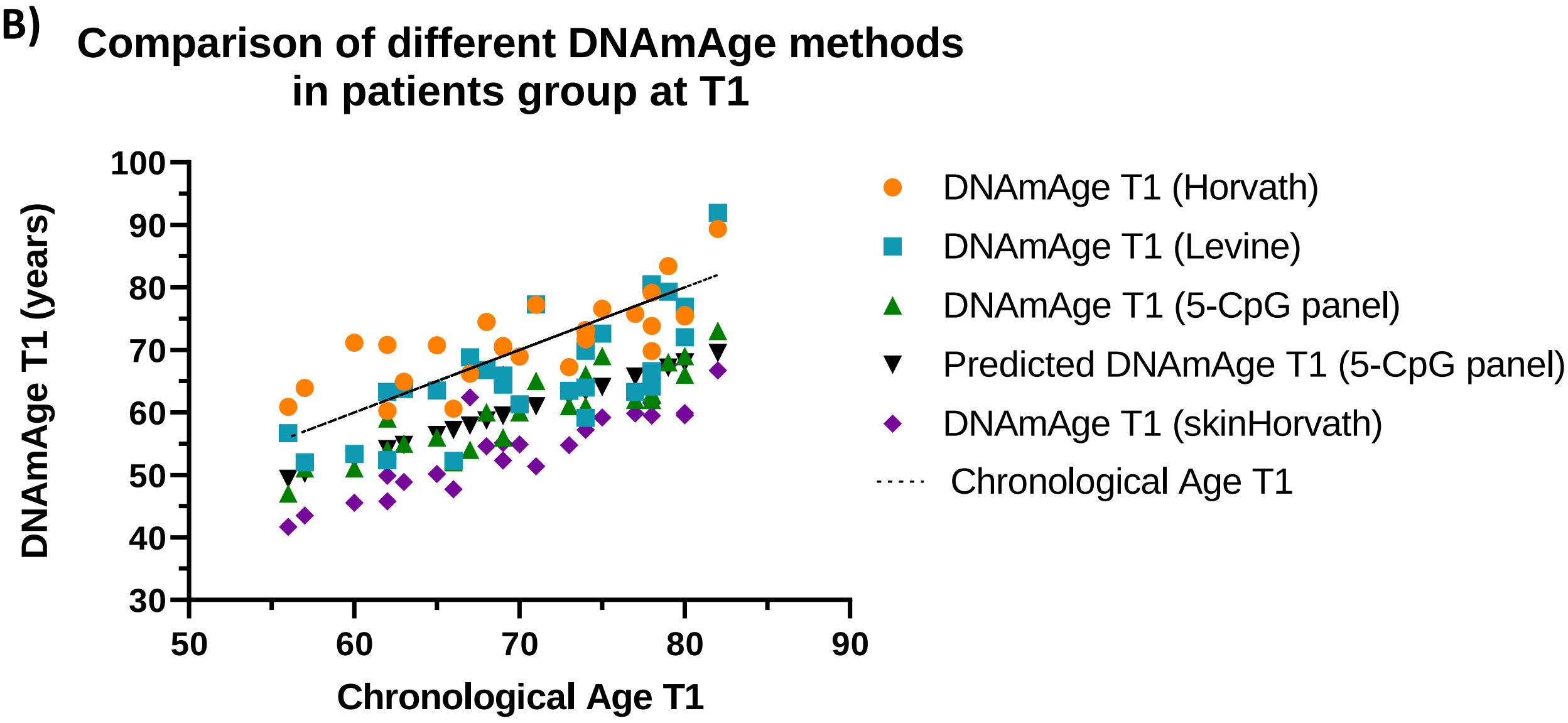

(A) DNAmAge estimates in IPF patients at baseline (T0) using the Horvath clock (Pearson r=0.781, p<0.0001), Levine clock (PhenoAge) (r=0.728, p<0.0001), 5-CpG panel DNAmAge (r=0.895, p<0.0001), predicted 5-CpG panel DNAmAge (r=1.000, p<0.0001) and Horvath Skin & Blood clock (r=0.850, p<0.0001). (B) DNAmAge estimates in IPF patients at follow-up (T1) using the Horvath clock (Pearson r=0.756, p<0.0001), Levine clock (PhenoAge) (r=0.735, p<0.0001), 5-CpG panel DNAmAge (r=0.846, p<0.0001), predicted 5-CpG panel DNAmAge (r=1.000, p<0.0001) and Horvath Skin & Blood clock (r=0.905, p<0.0001). Dotted lines represent identity lines corresponding to chronological age.

Supplementary Figure 2. Distribution of effect allele (EA) counts per individual among the 48 IPF patients.

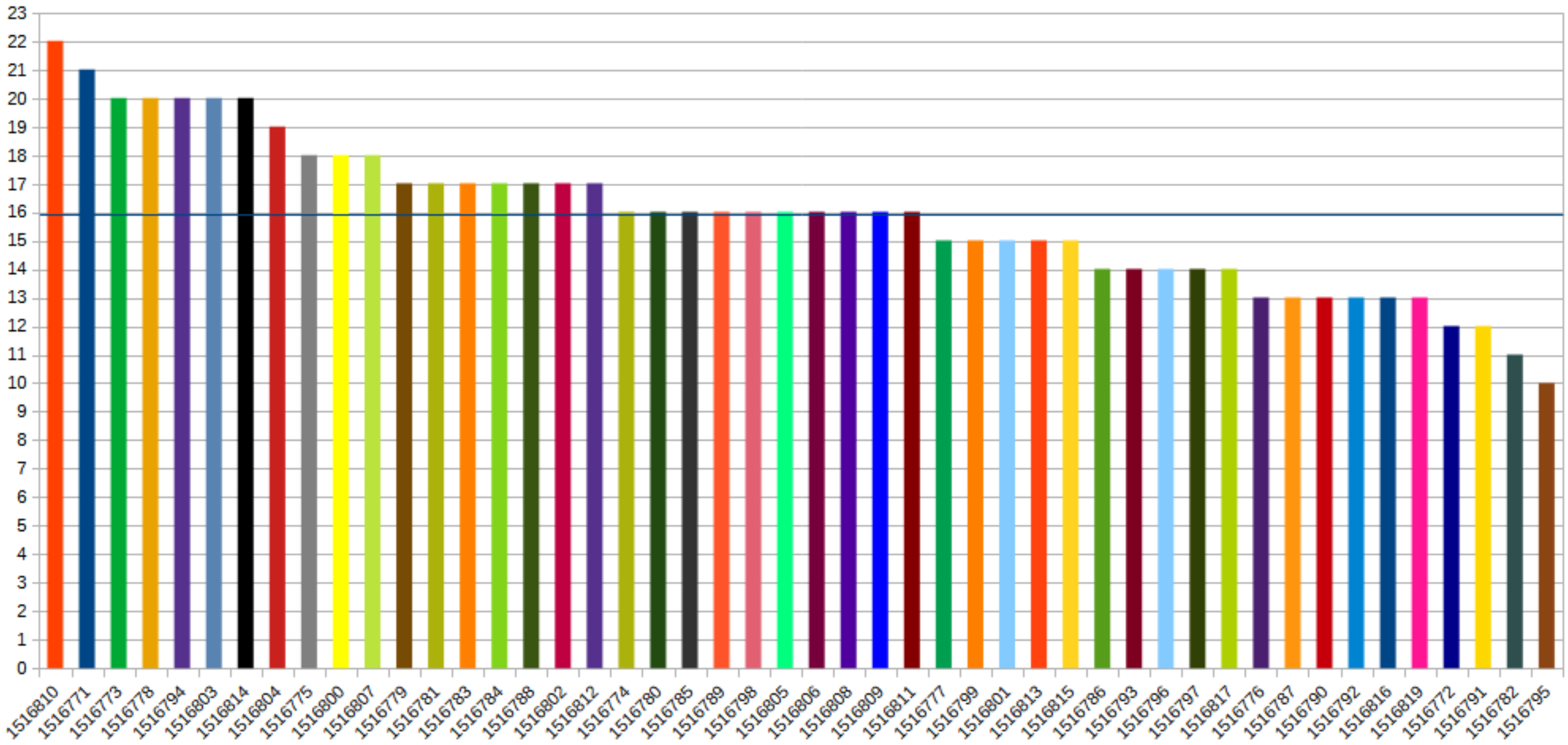

The chart plots the distribution of EA counts per individual among the IPF cohort (n=48 patients). Each bar represents the total number of EA carried by a single subject across the 17 SNPs (rs) analyzed. The number of EA ranged from 10 to 22, with a cohort mean of 16 (horizontal blue line), indicating inter-individual variability and potential differences in genetic susceptibility profiles.

Supplementary Figure 3. Targeted polygenic risk score (PGS004695) analysis in IPF.

(A)

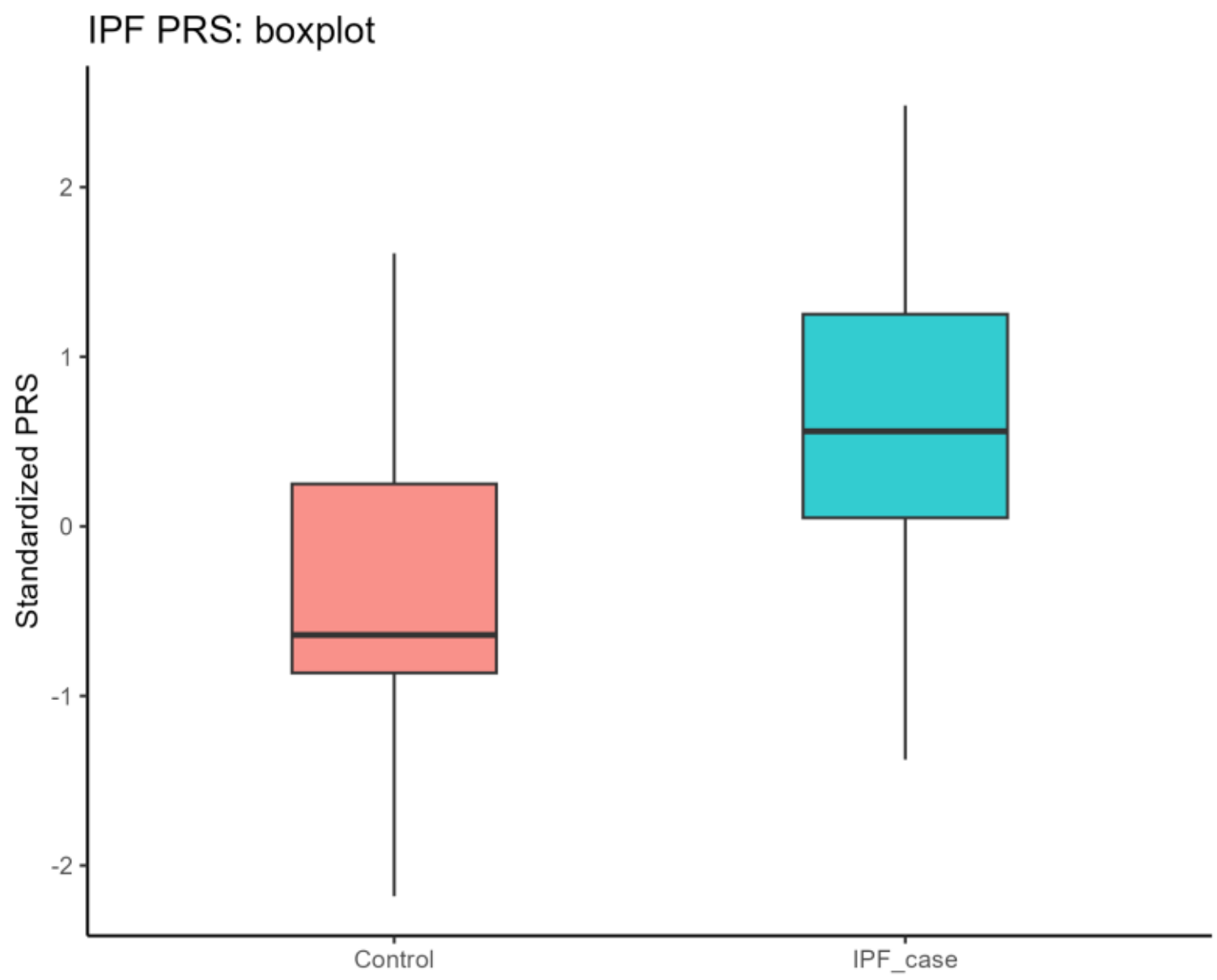

(B)

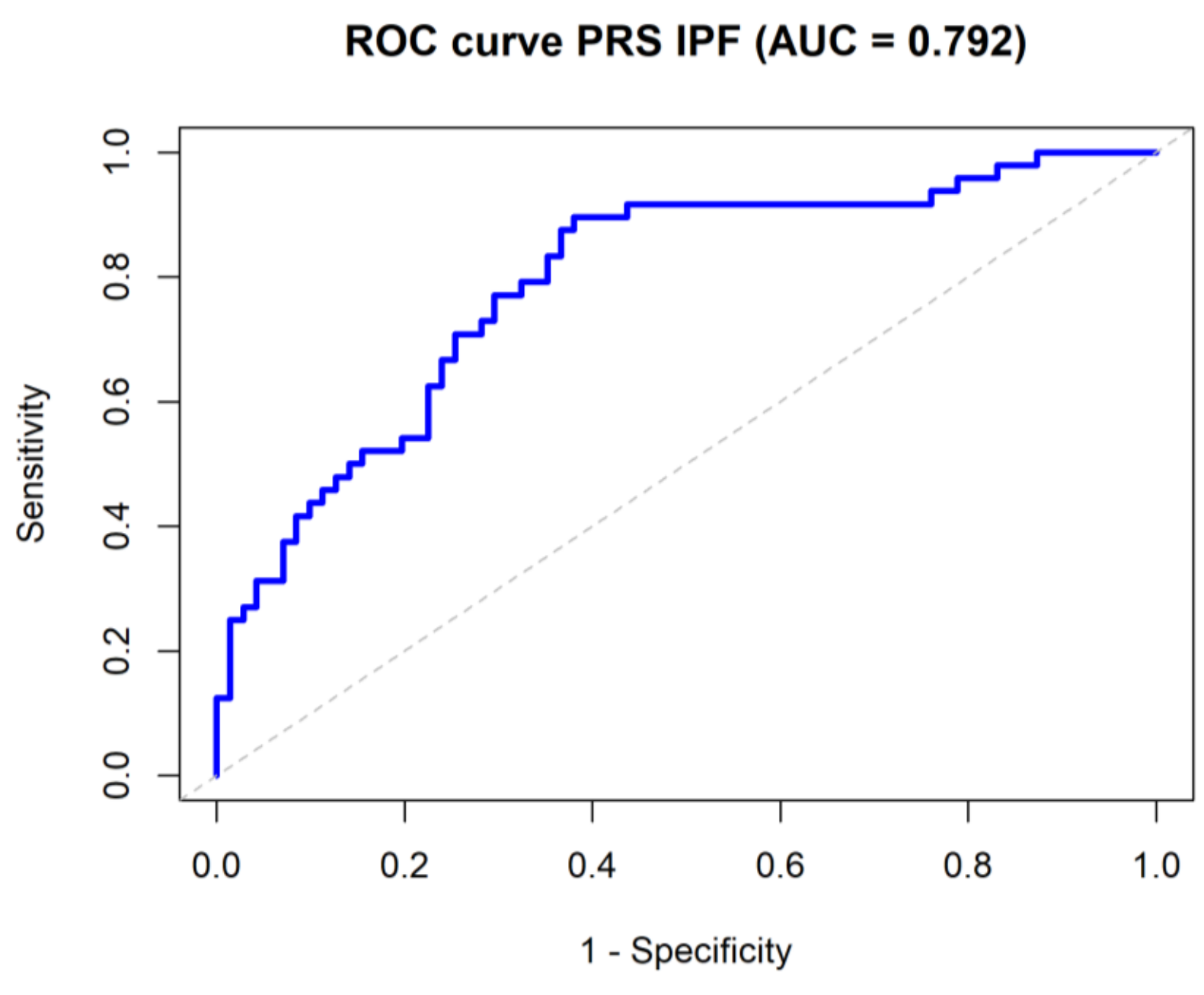

(A) Distribution of standardized targeted PRS (PGS004695) in IPF patients (n = 48) and healthy controls (n = 71), showing significantly higher values in cases compared to controls.
(B) Receiver operating characteristic (ROC) curve illustrating the discriminative performance of the PRS model, with an area under the curve (AUC) of approximately 0.79.
These results confirm that the targeted PRS captures a consistent genetic signal associated with IPF, in agreement with the genome-wide PRS model shown in Figure 8.

References

1. Fingerlin, T. E. *et al.* Genome-wide association study identifies multiple susceptibility loci for pulmonary fibrosis. *Nat Genet* **45**, 613–620 (2013).
2. Allen, R. J. *et al.* Genome-wide association study across five cohorts identifies five novel loci associated with idiopathic pulmonary fibrosis. (2022) doi:10.1136/thoraxjnl-2021-218577.
3. Mustafin, R. N. Molecular genetics of idiopathic pulmonary fibrosis. *Vavilovskii Zhurnal Genet Selektsii* **26**, 308–318 (2022).
4. Levy, D. *et al.* Genome-wide association identifies OBFC1 as a locus involved in human leukocyte telomere biology. *Proceedings of the National Academy of Sciences* **107**, 9293–9298 (2010).
5. Allen, R. J. *et al.* Genome-Wide Association Study of Susceptibility to Idiopathic Pulmonary Fibrosis. *Am J Respir Crit Care Med* **201**, 564–574 (2020).
6. Michalski, J. E. & Schwartz, D. A. <p>Genetic Risk Factors for Idiopathic Pulmonary Fibrosis: Insights into Immunopathogenesis</p>. *JIR* **13**, 1305–1318 (2021).
7. Lee, M.-G. & Lee, Y. H. A meta-analysis examining the association between the MUC5B rs35705950 T/G polymorphism and susceptibility to idiopathic pulmonary fibrosis. *Inflamm Res* **64**, 463–470 (2015).
8. Allen, R. J. *et al.* Genetic variants associated with susceptibility to idiopathic pulmonary fibrosis in people of European ancestry: a genome-wide association study. *The Lancet Respiratory Medicine* **5**, 869–880 (2017).
9. Noth, I. *et al.* Genetic variants associated with idiopathic pulmonary fibrosis susceptibility and mortality: a genome-wide association study. *Lancet Respir Med* **1**, 309–317 (2013).
10. Oldham, J. M. *et al.* PCSK6 and Survival in Idiopathic Pulmonary Fibrosis. *Am J Respir Crit Care Med* **207**, 1515–1524 (2023).

STROBE Statement—Checklist of items that should be included in reports of ***cohort studies***

	Item No	Recommendation	Page No
**Title and abstract**	1	(*a*) Indicate the study's design with a commonly used term in the title or the abstract	1
		(*b*) Provide in the abstract an informative and balanced summary of what was done and what was found	1
**Introduction**			
Background/rationale	2	Explain the scientific background and rationale for the investigation being reported	2-3
Objectives	3	State specific objectives, including any prespecified hypotheses	3
**Methods**			
Study design	4	Present key elements of study design early in the paper	9
Setting	5	Describe the setting, locations, and relevant dates, including periods of recruitment, exposure, follow-up, and data collection	9
Participants	6	(*a*) Give the eligibility criteria, and the sources and methods of selection of participants. Describe methods of follow-up	9
		(*b*) For matched studies, give matching criteria and number of exposed and unexposed	5
Variables	7	Clearly define all outcomes, exposures, predictors, potential confounders, and effect modifiers. Give diagnostic criteria, if applicable	9
Data sources/ measurement	8*	For each variable of interest, give sources of data and details of methods of assessment (measurement). Describe comparability of assessment methods if there is more than one group	9-11
Bias	9	Describe any efforts to address potential sources of bias	11
Study size	10	Explain how the study size was arrived at	11
Quantitative variables	11	Explain how quantitative variables were handled in the analyses. If applicable, describe which groupings were chosen and why	9-11
Statistical methods	12	(*a*) Describe all statistical methods, including those used to control for confounding	11
		(*b*) Describe any methods used to examine subgroups and interactions	9-11
		(*c*) Explain how missing data were addressed	n.a.
		(*d*) If applicable, explain how loss to follow-up was addressed	9, 11
		(*e*) Describe any sensitivity analyses	11
**Results**			
Participants	13*	(a) Report numbers of individuals at each stage of study—eg numbers potentially eligible, examined for eligibility, confirmed eligible, included in the study, completing follow-up, and analysed	3, Figure1
		(b) Give reasons for non-participation at each stage	3, 9
		(c) Consider use of a flow diagram	Figure 1
Descriptive data	14*	(a) Give characteristics of study participants (eg demographic, clinical, social) and information on exposures and potential confounders	3, Table 1, Table 2

		(b) Indicate number of participants with missing data for each variable of interest	3-5, Tables and Figures
		(c) Summarise follow-up time (eg, average and total amount)	3
Outcome data	15*	Report numbers of outcome events or summary measures over time	3-5, Tables 1-7, Figure 1-7
Main results	16	(*a*) Give unadjusted estimates and, if applicable, confounder-adjusted estimates and their precision (eg, 95% confidence interval). Make clear which confounders were adjusted for and why they were included	3-5, Figures, Supplementary information
		(*b*) Report category boundaries when continuous variables were categorized	11, Tables
		(*c*) If relevant, consider translating estimates of relative risk into absolute risk for a meaningful time period	11, 12, Tables
Other analyses	17	Report other analyses done—eg analyses of subgroups and interactions, and sensitivity analyses	Supplementary information
**Discussion**			
Key results	18	Summarise key results with reference to study objectives	5-9
Limitations	19	Discuss limitations of the study, taking into account sources of potential bias or imprecision. Discuss both direction and magnitude of any potential bias	8-9
Interpretation	20	Give a cautious overall interpretation of results considering objectives, limitations, multiplicity of analyses, results from similar studies, and other relevant evidence	5-9
Generalisability	21	Discuss the generalisability (external validity) of the study results	12
**Other information**			
Funding	22	Give the source of funding and the role of the funders for the present study and, if applicable, for the original study on which the present article is based	12

*Give information separately for exposed and unexposed groups.

Note: Information on the STROBE Initiative is available at http://www.strobe-statement.org.
